# Pan-Genome of the Genus *Streptomyces* and Prioritization of Biosynthetic Gene Clusters With Potential to Produce Antibiotic Compounds

**DOI:** 10.3389/fmicb.2021.677558

**Published:** 2021-09-28

**Authors:** Carlos Caicedo-Montoya, Monserrat Manzo-Ruiz, Rigoberto Ríos-Estepa

**Affiliations:** ^1^ Grupo de Bioprocesos, Departamento de Ingeniería Química, Universidad de Antioquia (UdeA), Medellín, Colombia; ^2^ Departamento de Biología Molecular y Biotecnología, Instituto de Investigaciones Biomédicas, Universidad Nacional Autónoma de México, Ciudad de México, Mexico

**Keywords:** pan-genome, *Streptomyces*, genome mining, comparative genomics, biosynthetic gene cluster

## Abstract

Species of the genus *Streptomyces* are known for their ability to produce multiple secondary metabolites; their genomes have been extensively explored to discover new bioactive compounds. The richness of genomic data currently available allows filtering for high quality genomes, which in turn permits reliable comparative genomics studies and an improved prediction of biosynthetic gene clusters (BGCs) through genome mining approaches. In this work, we used 121 genome sequences of the genus *Streptomyces* in a comparative genomics study with the aim of estimating the genomic diversity by protein domains content, sequence similarity of proteins and conservation of Intergenic Regions (IGRs). We also searched for BGCs but prioritizing those with potential antibiotic activity. Our analysis revealed that the pan-genome of the genus *Streptomyces* is clearly open, with a high quantity of unique gene families across the different species and that the IGRs are rarely conserved. We also described the phylogenetic relationships of the analyzed genomes using multiple markers, obtaining a trustworthy tree whose relationships were further validated by Average Nucleotide Identity (ANI) calculations. Finally, 33 biosynthetic gene clusters were detected to have potential antibiotic activity and a predicted mode of action, which might serve up as a guide to formulation of related experimental studies.

## Introduction

*Streptomyces* is the most prolific genus of the phylum *Actinobacteria* in terms of secondary metabolites production with high societal impact. It is estimated that members of the genus *Streptomyces* produce more than 50% of bioactive compounds produced by bacteria (Doroghazi and Metcalf, 2013). The variety of bioactive compounds produced by this genus include, among others, antifungals (e.g., amphotericin B by *Streptomyces nodosus*), anti-parasitic (e.g., avermectins by *Streptomyces avermitilis*), antivirals (e.g., virantmycin by *Streptomyces nitrosporeus*), immunosuppressant (e.g., rapamycin by *Streptomyces hygroscopicus* and other strains), chemotherapeutics (e.g., daunorubicin by *Streptomyces peucetius*), and a wide variety of antibiotics as tetracycline produced by *Streptomyces rimosus* and streptomycin produced by *Streptomyces griseus* (Nakagawa et al., 1981; Pham et al., 2019).

The advent of next-generation sequencing technologies unveiled the metabolic potential of bacteria as producers of secondary metabolites. *Streptomyces coelicolor*, for instance, only produces actinorhodin, undecylprodigiosin, calcium-dependent antibiotic and methylenomycin at laboratory conditions, though its genome contains over 20 biosynthetic gene clusters (Challis and Hopwood, 2003). The rapid progress on genomic sequencing and the decrease in sequencing prices have enabled obtaining a vast quantity of genomes that has led to a deeper knowledge of microorganisms capable of synthesizing bioactive compounds, and the discovery of biosynthetic gene clusters that might produce novel compounds with clinical and commercial value (Kalkreuter et al., 2020).

The analysis of such amount of genomic data is a challenge; nevertheless, it may pave the way for performing comparative genomics studies, which help revealing the microbial diversity of a genus, genes involved in environmental adaptations, antibiotic resistance, and genes that confer the ability to colonize novel niches (Tettelin et al., 2008; Niu, 2018). Previous comparative genomic studies in the genus *Streptomyces* showed the genetic variability and the biosynthetic potential of the genus (Jackson et al., 2018; Xu et al., 2019; Belknap et al., 2020; Lee et al., 2020); genes involved in osmotic stress defense, symbiotic interactions, among other environmental niche adaptation genes were identified in marine *Streptomyces* (Tian et al., 2016; Almeida et al., 2019).

These reports, however, include only a few *Streptomyces* strains or a mix of complete and incomplete genomes that could render unreliable results. In this study, we present the first comparative genomic study for the genus *Streptomyces* with a large amount of complete and high-quality genomes available, unveiling the pan-genome in terms of protein sequence similarity and protein domains content, describing phylogenetic relationships of the analyzed genomes, as well as highlighting the variability of their intergenic regions (IGRs) and their capability of producing bioactive compounds; the study prioritizes in the biosynthetic gene clusters (BGCs) with potential antibiotic activity and a predicted mode of action according to the co-localization of duplicated self-resistance genes.

## Materials and Methods

### Selection of Genomes

For the present study, the genomes were selected based on their quality and completeness. All genomes of the genus *Streptomyces* with status “Complete Genomes” were downloaded from Reference Sequence (Refseq; December 2019). After manual curation, 121 high quality genomes were included for the subsequent analysis. We further evaluated the genome assembly quality through the determination of genome completeness with BUSCO against the lineage dataset *streptomycetales_odb10*, which contains 145 species and 1,579 BUSCOs (Simão et al., 2015).

### Pan-Genome Estimation

Genomes were downloaded from RefSeq in *genbank* and *faa* formats. We executed Roary to calculate the pan-genome for the genus *Streptomyces* (Page et al., 2015). Previously, we evaluated other software for pan-genomics studies such as BPGA (Chaudhari et al., 2016), GET_HOMOLOGUES (Contreras-Moreira and Vinuesa, 2013), Micropan in mode *blast all vs all* (Snipen and Liland, 2015) and Roary. The latter was selected because it had one of the lowest running times and generates the output for conservation of intergenic regions analysis, while producing similar results to the other tools. Roary requires genomes in gff3 format along with the sequences at the end of the file. To produce such files, we converted the *genbank* files using the BioPerl script *bp_genbank2gff3.pl* (McKay, 2004). The minimum percentage identity for BLASTp searches was set to 70%; the splitting of paralogs was blocked because it was required for the determination of the conserved IGRs. The maximum number of clusters was adjusted to 170,000. An alignment of core genes detected by Roary was created using MAFFT (Katoh and Standley, 2013) and we utilized this alignment to build a maximum likelihood phylogenetic tree using the software FastTree2 (Price et al., 2010) implemented in the Galaxy Europe server (Goecks et al., 2010).

In addition, we characterized the pan-genome of the genus *Streptomyces* based on the domain diversity of proteins encoded in the analyzed genomes. We used the R package Micropan version 1.2 (Snipen and Liland, 2015). Briefly, all the amino acid sequences of encoded proteins of the 121 genomes were annotated for their domain content with HMMER 3.3.1 (Eddy, 2011) against the Pfam-A database (Finn et al., 2014). Clustering made by Micropan is based on the presence of domains; thus, proteins sharing the same domains were grouped in the same gene family or cluster. The function BionomixEstimate of Micropan was implemented to extrapolate the size of the pan-genome using the presence/absence matrix resulting from both previous analyses. For both methodologies, we also determined the fluidity and the Jaccard distance for the genomes of the streptomycetes using the corresponding functions in Micropan.

All genes were classified as core, soft-core, shell, and cloud genes according to their presence among the genomes analyzed. Thus, genes present in the 121 strains were designated as core genes; genes present in more than 95% of strains (115 strains) were classified as soft-core genes; shell genes were those with a presence between 15 and 95% (19 and 114 strains), and genes present in less than 15% of the strains analyzed (less than 19 genomes) were assigned as cloud genes. For both methodologies, Roary and Micropan, we extracted representative sequences of the core, soft-core, shell, and cloud genes with in-house built Biopython scripts for subsequent functional annotation.

Genes resulting from Roary were translated into amino acid sequences; then, functional description of pan-genome categories defined for both methodologies was carried out determining gene ontology (GO) terms for the selected proteins (The Gene Ontology Consortium, 2019). This was performed with the Interproscan functional predictions of ORFs tool available in the Galaxy Europe Server (Quevillon et al., 2005). The results were summarized and plotted in WEGO 2.0 (Ye et al., 2018). Additional annotations were obtained through the WebMGA server for Clusters of Orthologous Groups (COG) assignments (Wu et al., 2011). Finally, the phylogenetic tree built with core genes along with information of the habitat and number of genes for each pan-genome category were visualized with Itol (Letunic and Bork, 2019).

### Conservation of Intergenic Regions

In agreement with the phylogenetic tree, we defined three groups to analyze the conservation of IGRs in more closely related organisms; *Streptomyces xiamenensis* 318 and *Streptomyces cattleya* NRRL 8057 were left out of this analysis since no obvious relation with other *Streptomyces* was found. We estimated the conservation of intergenic regions across the streptomycetes using the software Piggy (Thorpe et al., 2018). The results of the previous analysis in Roary were used as input for Piggy. The software parameters *nuc_id* and *len_id* were set to 70, which is in accordance with the values used in Roary. Default values were used for the other parameters. Following this procedure, we analyzed the IGRs in the predefined groups of *Streptomyces*. The parameters used to analyze the groups of genomes were the same for the analysis of the complete set of genomes. Moreover, we aligned the IGRs conserved in more than 90% of the genomes included in each group against Rfam (version 14.5) database (Kalvari et al., 2021). Then, we explored for possible non-coding RNAs presence in these conserved IGRs with the software RNAz (Gruber et al., 2010). We previously filtered the IGRs alignments with the command *rnazSelectSeqs.pl* to preserve the sequences with a mean pairwise identity of 70%. Only the outputs with an overall RNA-class probability above 0.7 were considered as putative non-coding RNAs; their secondary structures were visualized with RNAfold (Lorenz et al., 2011) and their possible targets were defined using IntaRNA 2.0 (Mann et al., 2017).

### Phylogenomic Analysis

The Galaxy wrapper of fastANI, with default parameters and using an all-*versus*-all genome comparisons, was implemented to calculate the average nucleotide identity (ANI) for the 121 selected strains (Jain et al., 2018). The heat map and dendrogram for the results of fastANI were generated using the libraries Seaborn and Matplotlib of Python (Hunter, 2007). The linkage method was the UPGMA algorithm and the pairwise distances between observations was the Euclidean metric. For those genomes with ANI values higher than 95% and with ambiguous taxonomic affiliations, we performed global genome alignments with progressiveMauve (Darling et al., 2010).

### BGCs Prediction, Prioritization, and Similarity Comparison

All 121 genomes were analyzed using ARTS 2.0 (available at https://arts.ziemertlab.com) with default settings. ARTS 2.0 used antiSMASH 5.1.1 for BGCs prediction (Blin et al., 2019). Since the lack of a proper methodology to define gene cluster boundaries, antiSMASH outputs a series of biosynthetic gene cluster regions; each region can be comprised by one or more co-localized “candidate” clusters; each “candidate” cluster defined by antiSMASH contains the biosynthetic machinery to produce a type of metabolite. In this work, we call BGC to each “candidate” cluster (for more information of antisSMASH definitions see: https://docs.antismash.secondarymetabolites.org/understanding_output/). After running the antiSMASH analysis, ARTS identifies BGCs co-localized with self-resistance enzymes (based on Resfam database), and with core genes (defined by ARTS using a database of actinomycetes genomes) with predicted horizontal gene transfer (HGT; Alanjary et al., 2017; Mungan et al., 2020). All predicted clusters of our interest were searched in the repository of the Minimum Information about a Biosynthetic Gene Cluster (MIBiG) database (Kautsar et al., 2020; available at https://mibig.secondarymetabolites.org).

The 3,750 *genbank* files, derived from the antiSMASH analysis, and the 33 *genbank* files corresponding to the BGCs prioritized by ARTS 2.0, were used as input for BGC similarity comparison using BiG-SCAPE 1.1.2 (Navarro-Muñoz et al., 2020). Analyses were made setting cutoff values at 0.3, 0.5, and 0.7 with and without the MIBiG database. Results of the networks, including the MIBiG database, were then filtered to remove comparisons between BGCs from the MIBiG database that did not display similarity with clusters from our analysis. Similarity comparison matrices were visualized using Cytoscape 3.8.2 (Shannon et al., 2003).

## Results

### General Features of *Streptomyces* Genomes

High quality genomes were included in the present investigation; apart from the status as “Complete genomes,” all genomes showed a high completeness and a reduced number of fragmented and missing genes (Supplementary Figure S1). Genome size ranges from 5.96 Mb for *Streptomyces xiamenensis* 318 to 12.01 Mb for *S. hygroscopicus* XM201; both strains also contain the minimum and the maximum protein coding genes with 5,100 and 9,385, respectively. The %G+C mean content is 71.77 +/− 0.81, which is an expected characteristic of members of the phylum *Actinobacteria* (Supplementary Figure S2; Dhakal et al., 2017). Most strains have a unique chromosome, although notably, the strain *S. hygroscopicus limoneus* KCTC 1717 has two chromosomes (Lee et al., 2016); all strains contain between one and four plasmids. Supplementary File S1 comprises all the metadata collected i.e., it contains the information of genome accession numbers, sequencing platform, coverage, and other genomic features such as the number of tRNAs and rRNAs in each genome.

### Comparative Genomics of the Genus *Streptomyces* Through Clustering of Protein Sequences by Similarity and Domains Content

**Figure 1:**
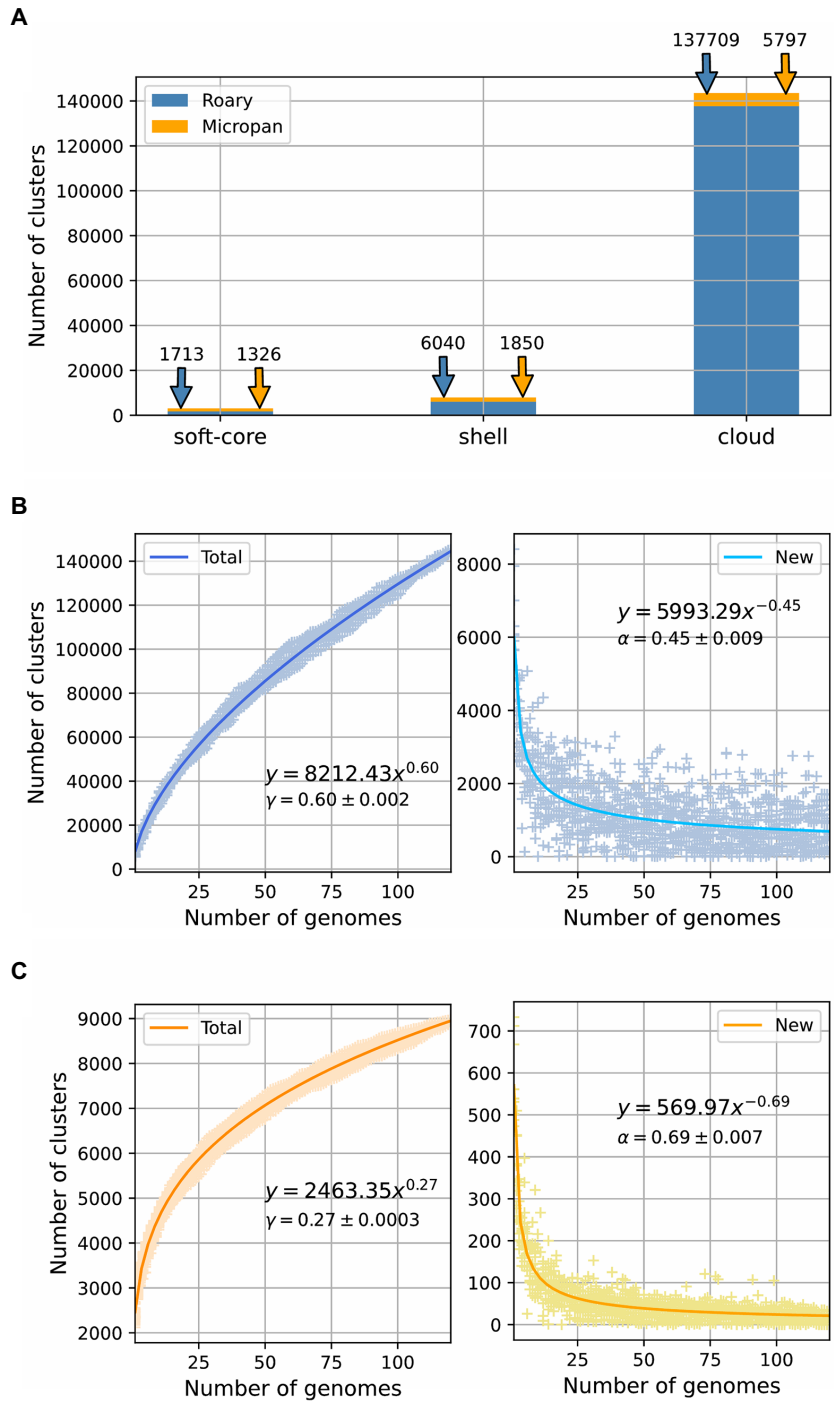
Pan-genome estimation for the genus *Streptomyces*. **(A)** Pan-genome categories size for calculations using Roary and Micropan; “soft-core” label includes both core and soft-core genome. Power law fit for the number of total genes and new genes as a function of the number of genomes added to the analysis for **(B)** Roary and **(C)** Micropan.

We determined the pan-genome of the genus *Streptomyces* to establish their microbial diversity in terms of protein coding genes, domains content, and regulatory elements located in intergenic regions. For this purpose, we used different methodologies to accurately represent its entire gene repertoire. The analysis with Roary, to determine the diversity of protein coding genes, showed that the pan-genome of *Streptomyces* is clearly open (*α*<1, 0<*γ*<1) with a size of 145,462 clusters (Figures 1A,B). By using the BionomixEstimate function of Micropan, the current data allowed extrapolation to a total size of 273,372 clusters. These clusters were then classified according to their conservation level among the genomes analyzed. In concordance with this classification, we obtained 633 core genes, 1,080 soft-core genes, 6,040 shell genes, and 137,709 cloud genes; interestingly in the last group 81,568 were unique clusters, which means they were only present in one genome among all the considered strains.

Micropan estimated the pan-genome size of the genus *Streptomyces* as 8,973 protein families or clusters. Although, this number is clearly low compared to that determined by Roary, the power law fit, performed for the total size of the pan-genome and the number of new genes, showed that the pan-genome was still open (*α*<1, 0<*γ*<1), even though, it was set in the boundaries of a close pan-genome, as the value of the gamma parameter was close to zero (Figure 1C). The BionomixEstimate function, applied to both methods, displayed a similar core genome size of 600 and 589 for Roary and Micropan, respectively. Figure 1 summarizes the pan-genome calculations.

The fluidity of the pan-genome, which determines how dissimilar genomes are at a gene level, was estimated for both procedures employed to assess the genomic diversity of the genus *Streptomyces* (Kislyuk et al., 2011). The fluidity value was 0.53 +/− 0.099 for Roary and 0.22 +/− 0.031 for Micropan. This indicates that *Streptomyces* genomes differ 53%, on average, if the similarity of protein sequences are used to build the pan-genome, and 22% if their domain distributions are considered. A related assessment of genome diversity can be performed by the Jaccard distance distribution (Jaccard, 1912), which is roughly defined as one minus the number of genes shared by two genomes, divided by the total number of genes in these two genomes; the higher the value of Jaccard distance the more dissimilar the two genomes are. Overall, the Jaccard distance for both methodologies, Roary and Micropan, displayed similar distributions (Supplementary Figures S3A,B, respectively), centered at different mean values. Thus, highly similar genomes, as those genomes of the same species, possess the same genes/domains frequency giving values close to zero.

### Phylogenomic Analysis

One of the main outcomes of a pan-genomic study is the determination of the genes shared by all members of a determined group, which corresponds to the core genome, previously defined. These core genes can be concatenated and aligned to define phylogenetic relationships among the members of a group, as this approach possesses higher resolution than using a single phylogenetic marker, e.g., 16S rRNA gene; thus, it has been suggested as the basis for bacterial phylogeny (Parks et al., 2018). Furthermore, a combination of results of multiple markers, such as the core genome phylogeny, above mentioned, and overall genome relatedness indices (from which ANI is the most broadly used), has been proposed to obtain precise taxonomic affiliations (Figueras et al., 2014). In this regard, we used both approaches, to explore the phylogeny in the genus *Streptomyces*.

**Figure 2:**
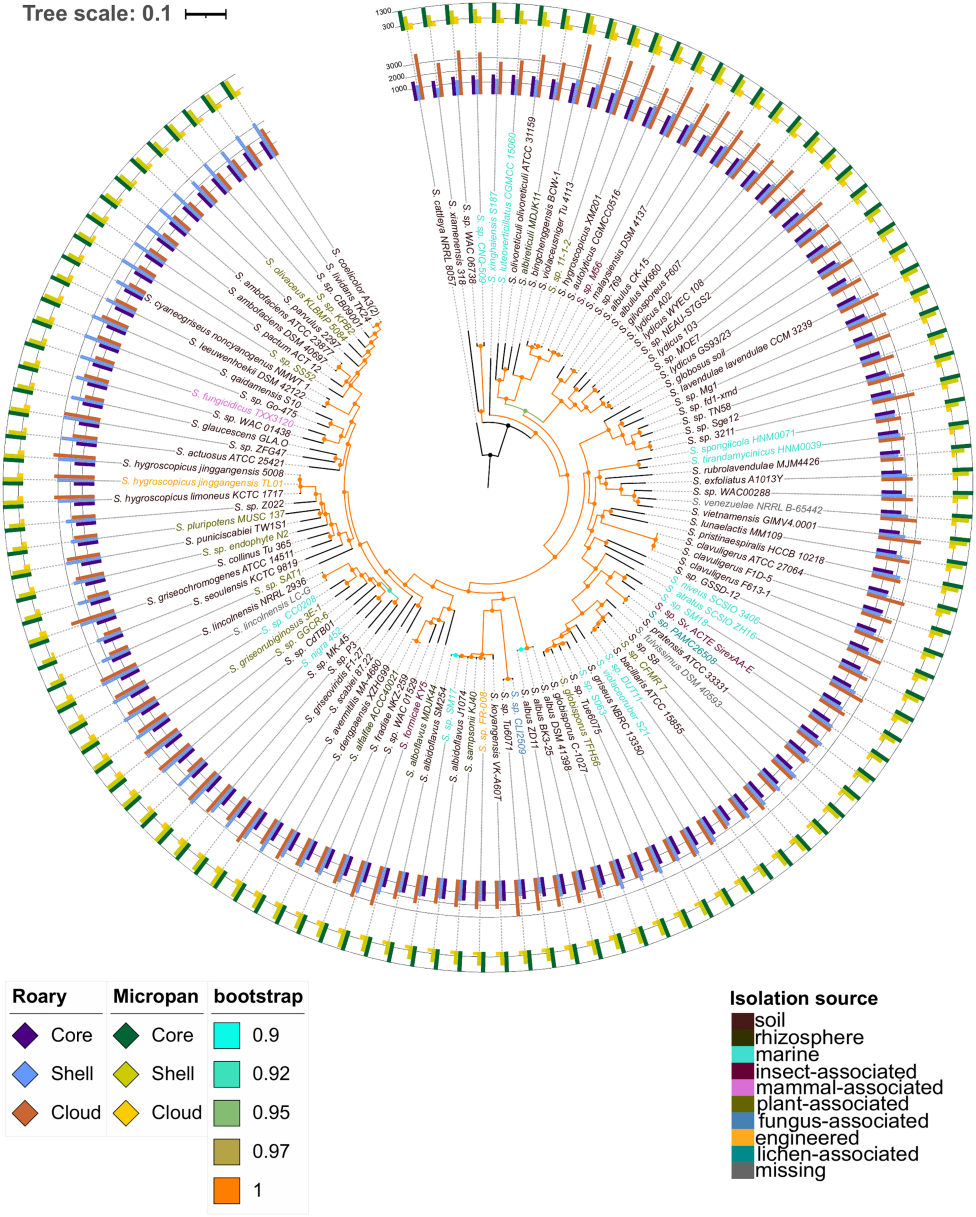
Core genome phylogenetic tree constructed using conserved genes across all genomes considered. Color codes are included for the bootstrap support and the isolation source of the microorganisms. The inner bars represent the number of genes in each strain belonging to the core and soft-core genome, (labeled as core), shell genome, and cloud genome for the pan-genome determination using Roary. The outer bars represent the number of genes in each strain belonging to the core and soft-core genome, (labeled as core), shell genome, and cloud genome according to the analysis in Micropan.

Alignment of a set of 633 core genes, calculated by Roary, allowed the construction of a high confidence phylogenetic tree. The bootstrap values for all branches were above 0.9 being the majority equal to 1 (Figure 2). The number of genes in each genome, that are part of the different pan-genome categories, are also depicted in this figure, depending on the method used to determine the pan-genome. Based on the core genome phylogenetic tree, there was no clear relationship between the isolation source of the strains and its evolutionary relationship with other strains. Three clades were clearly distinguishable in the phylogenetic tree; they are highlighted in Supplementary Figure S4, for the sake of clarity.

Interestingly, *S. hygroscopicus* XM201 was set in group 1, while the other *S. hygroscopicus* strains were in group 3; in addition, the XM201 strain was closer to *Streptomyces* sp. 11-1-2 and *S. violaceusniger* Tu 4113, whose ANI values were 98.5 and 95.5, respectively. Other genomic features such as the genome size and the number of proteins encoded in the genome were also similar among these strains. *Streptomyces lydicus* strains formed a paraphyletic taxon; *S. lydicus* A02 was closer to *S. gilvosporeus* F607 than to the other strains classified as *S. lydicus*; this outcome was supported by ANI values in a range of 86–89%. A similar result was found for *S. lydicus* WYEC 108 whose ANI values were between 86 and 88% with other *S. lydicus*, and 96.7% with *Streptomyces* sp. NEAU-S7GS2. Lastly, *Streptomyces* sp. MOE7 contained an ANI value of 97.8% with *S. lydicus* GS93/23. ANI values of *Streptomyces autolyticus* CGMCC0516, *Streptomyces malaysiensis* DSM 4137 and *Streptomyces* sp. M56 were above 98% among them, which could indicate that they are the same species. Other strains that showed high ANI values between species and close relationship in the phylogenetic tree of core genes were: *Streptomyces pratensis* ATCC 33331 and *Streptomyces* sp. PAMC26508 (99.1 ANI); *Streptomyces bacillaris* ATCC 15855, *Streptomyces* sp. DUT11, *Streptomyces* sp. CFMR7 and *Streptomyces* sp. S8 (ANI values between 95.7 and 98.9%); both strains of *Streptomyces globisporus* with *Streptomyces* sp. 6063 and *Streptomyces* sp. Tue6075 (ANI values greater than 95.1); *Streptomyces fradiae* NKZ-259 and *Streptomyces alfalfae* ACCC40021 with an ANI value above of 99.9%, which might suggest they are the same strain, though further experimental studies are vital to prove it. The strains VK-A60T, KJ40, Fr-008, J1074, SM254, and SM17 all have ANI values greater than 95.8% among them.

An interesting clade is the one formed by *Streptomyces* sp. CB09001, the model organism *Streptomyces coelicolor* A3(2) and the biotechnologically important actinobacteria *Streptomyces lividans* TK24. A comparison of these strains showed that the genome of *S. coelicolor* A3(2) is almost 0.8 Mb larger than the *S. lividans* TK24 genome and 1.2 Mb larger than the one of *Streptomyces* sp. CB09001. Thus, we performed a global alignment of the genomes of these species to corroborate the observed relationship (Supplementary Figure S5). This assessment showed that the *S. lividans* TK24 genome is a reduced version of the *S. coelicolor* A3(2) genome, which has an additional region of about 0.6Mb in one of the telomeres. These results are in consensus with a recent study indicating that the ANI value between *S. lividans* and *S. coelicolor* is 99.0% (Vicente et al., 2018). Surprisingly, all the results observed in the core genome phylogenetic tree are validated by the corresponding ANI values among the species clustered together. In addition, a deep analysis for a possible reclassification of some species is suggested by the outcomes of the present study. These results can be observed in the Figure 2 and Supplementary Figure S4 for the phylogenetic tree and in the Supplementary Figure S6 for ANI values.

### Intergenic Regions Conservation

**Figure 3:**
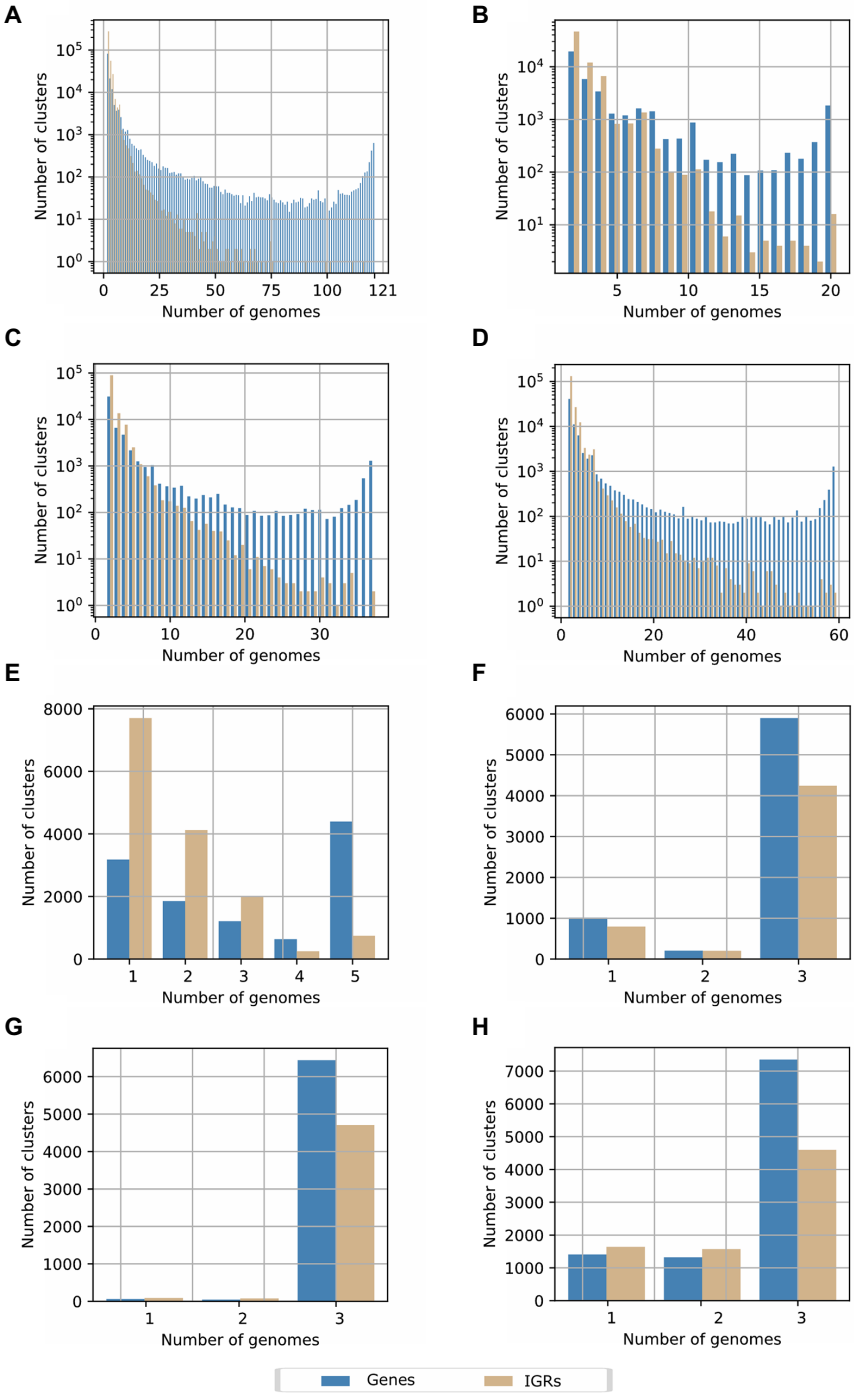
Pan-genome determination based on the conservation of intergenic regions (IGRs) in the *Streptomyces* genus for **(A)** all species included in the current study. **(B)** Species belonging to the clade or group 1 in the core genome phylogenetic tree. **(C)** Species belonging to the clade or group 2 in the core genome phylogenetic tree. **(D)** Species of the clade 3 in the core genome phylogenetic tree. **(E)** Strains of the paraphyletic group of *Streptomyces lydicus*. **(F)**. *Streptomyces clavuligerus* ATCC 27064, F1D-5 and F613-1. **(G)** *Streptomyces albus* DSM 41398, BK3-25 and ZD11. **(H)** *Streptomyces hygroscopicus* 5008, TL01, and KCTC 1717.

The number of IGRs was astonishingly high and variable, and no core group of IGRs was determined for the genus *Streptomyces*. The total number of IGR were 378,972; of these, 275,225 correspond to unique clusters of IGRs, which was more than twice the number of unique gene clusters obtained with Roary. As observed in Figure 3A, IGRs were only conserved across few strains. We further explored these results by analyzing the IGRs through the groups defined in the core genome phylogenetic tree, previously described (Supplementary Figure S4). In group 1, comprising 20 species (Figure 3B), the number of IGRs were still high (46,301 compared to 19,333 gene families), although, in this group there were 16 core IGRs. For group 2 (Figure 3C), which contained more species compared to group 1, only two IGRs were conserved in all 37 species belonging to this group; meanwhile, in group 3 (Figure 3D) integrated by 59 species, 131,213 clusters were unique IGRs and two were defined as core IGRs. Overall, IGRs clusters showed a pronounced drop as the number of genomes increased which differed from the behavior displayed for gene clusters, which were mainly unique or core genes (Figure 3A). Surprisingly, no annotations were retrieved from Rfam when representative sequences of these few conserved IGRs were searched. From these, 10, 1, 3 IGRs belonging to the groups previously defined, contain putative novel small or non-coding RNAs due to the conserved RNA secondary structures detected by RNAz (Supplementary File S2). The minimum free energy (MFE) structure of these predicted small RNAs (sRNAs) can be visualized in Supplementary Figures S7–S20. Additionally, we estimated the possible targets of these putative novel non-coding RNAs, in the genomes from which we extracted the representative IGRs sequences. Overall, we found six sequences that share full complementarity with the mRNA located down-stream, which suggests they can act as regulatory elements in the untranslated region of these genes; by other hand, multiple targets were detected that can interact with these sRNAs. The details of these analyses can be observed in the Supplementary File S2.

To investigate the IGRs conservation between more related *Streptomyces* species, we further analyzed the pan-genome of IGRs of *S. lydicus* (Figure 3E), *Streptomyces clavuligerus* (Figure 3F), *Streptomyces albus* (Figure 3G), and *S. hygroscopicus* (Figure 3H), as representatives of the three groups previously defined in the *Streptomyces* phylogeny. In the case of the paraphyletic group of *S. lydicus*, 248 IGR core clusters and 7,705 unique IGRs clusters were found. In *S. clavuligerus*, *S. hygroscopicus*, and *S. albus*, the number of core IGRs were 4,284, 4,597, and 4,706, respectively, which were considerably higher than the number of unique IGRs. This behavior agrees with the number of genes shared by the genomes, but it contrasts with the results obtained from the different groups of the phylogeny, and when all genomes of the genus were considered. Hence, we observed that IGRs are only conserved between phylogenetically related species.

### Functional Description of the Pan-Genome

**Figure 4:**
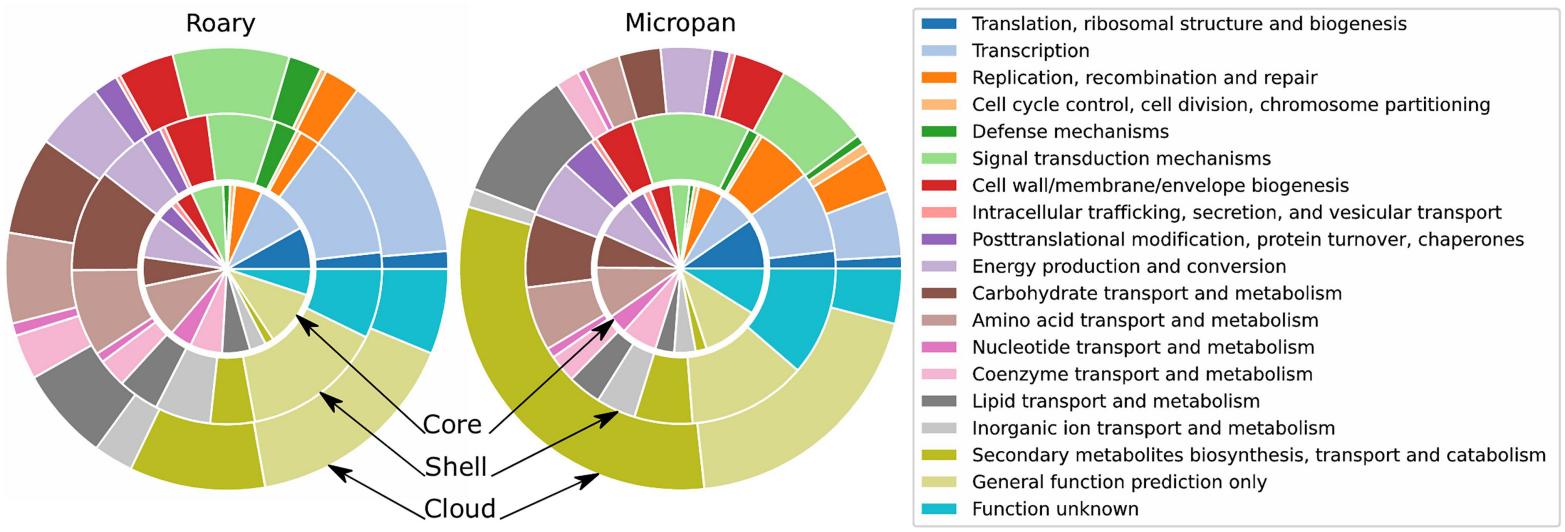
Functional description of the categories of the pan-genome for Roary and Micropan by means of Clusters of Orthologous Groups (COG) annotations. Inner circle represents the core and soft-core genomes, the middle circle represents the shell genome, and outer circle represents the cloud genome.

Genes of the acquired pan-genome were then functionally classified. The COG functional enrichment demonstrated that the most conserved genes and family of proteins are those involved in primary metabolism and DNA processing functions (Figure 4). Interestingly, the abundance of secondary metabolism genes increases in less conserved genes, i.e., cloud genes. This tendency is more evident in the Micropan analysis, where protein domains of secondary metabolite genes represent more than 25% of total protein domains in cloud genes; lipid metabolism, frequently used for secondary metabolites production (Liu et al., 2013), also predominates.

The analysis of GO categories displayed similar results. Primary metabolism and catalytic processes as organic cyclic and heterocyclic compound binding are over-represented in core genes analyzed by Roary (Supplementary Figure S21). The GO enrichment in genes analyzed using Micropan evidences the abundance of genes involved in cellular and metabolic processes, as well as the abundance of the catalytic activity genes in all levels of conservancy, denoting the catalytic power of the genus.

### BGCs Prediction and Prioritization

Genomes were analyzed using ARTS 2.0 to prioritize BGCs more likely to produce an active metabolite, based on the presence of self-resistance enzymes co-localized within BGCs, as well as the presence of duplicated core genes with evidence of HGT (Alanjary et al., 2017; Mungan et al., 2020).

The analysis with antiSMASH displayed 3,750 regions of BGCs (Supplementary File S3). Since some BGCs can be co-localized in the same region (up to nine BGCs in one region), we separated the BGCs afterward to do the final count. However, it is worthy to point out that some co-localized BGCs could act as hybrid clusters, such as the modular system NRPS/T1PKS, which is widely found in the three domains of life (Wang et al., 2014).

Overall, 5,289 BGCs were identified in the 121 genomes analyzed, distributed in the 3,750 regions. Per order of frequency, non-ribosomal peptide synthetase (NRPS), terpene, type 1 polyketide synthase (T1PKS), and siderophore were the predominant BGC types, accounting for almost 50% of total predicted BGCs. Each genome accounts for 23–83 BGCs (average=44, median=42), *Streptomyces griseochromogenes* ATCC 14511 carried 83 BGCs and *Streptomyces* sp. CLI2509 carried 23. The biosynthetic potential of *S. griseochromogenes* ATCC 14511 was already unveiled by Wu et al. (2017a).

**Figure 5:**
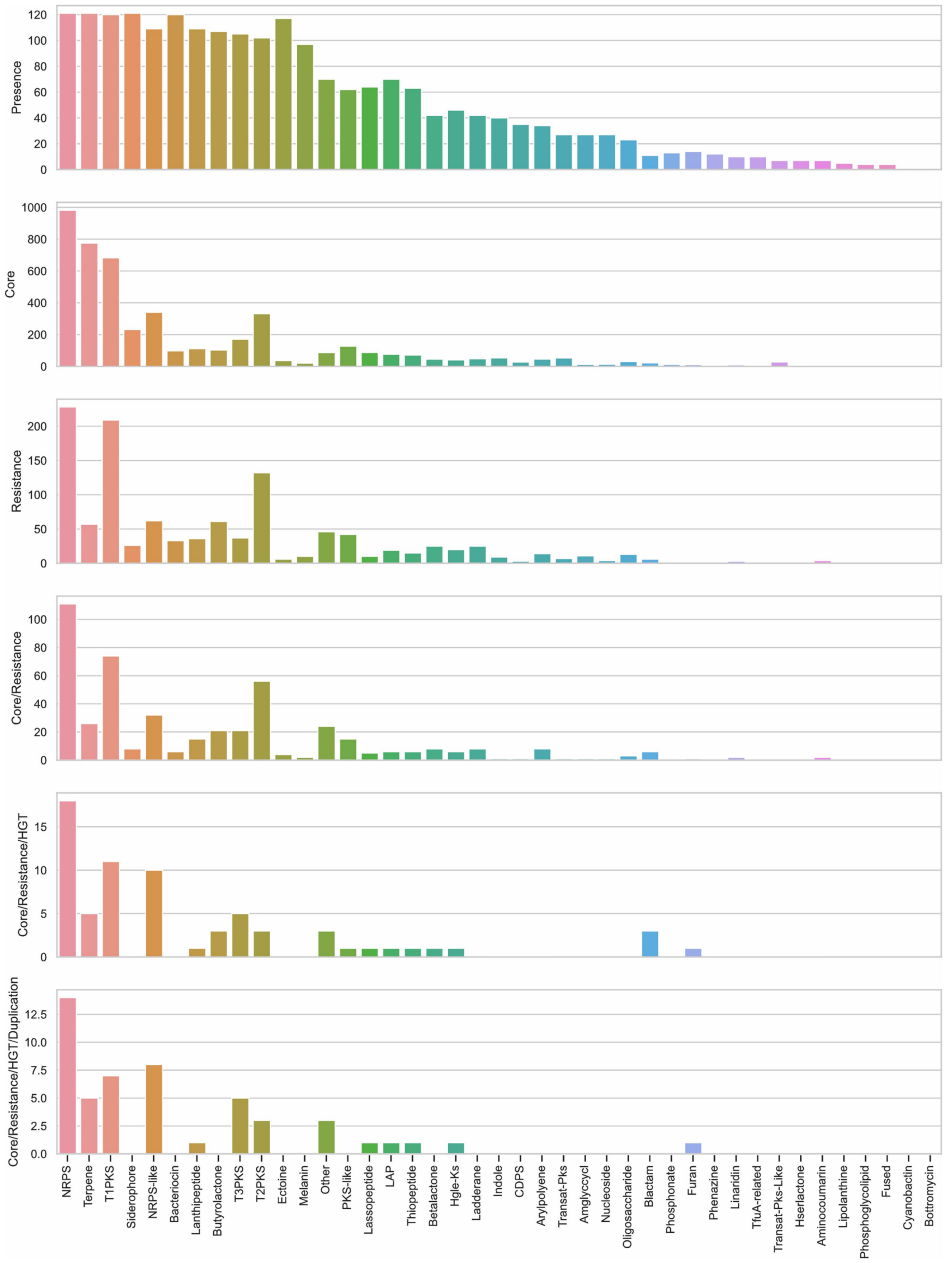
Description of biosynthetic gene clusters (BGCs) according to their presence among genomes, their proximity to core and self-resistance genes, and core genes with evidence of horizontal gene transfer (HGT) and duplication. Bar graph of presence shows the counting of BGCs present in the analyzed genomes. Bar graphs of core and resistance display the counting of core and self-resistance genes (resistance) located nearby the BGC. Core/Resistance graph shows the counting of BGCs co-localized with both core and self-resistance genes. Core/Resistance/HGT graph displays the counting of BGCs co-localized with self-resistance genes and core genes with evidence of HGT. Core/Resistance/HGT/Duplication chart shows the counting of BGCs co-localized with self-resistance genes and core genes with evidence of both HGT and duplication. NRPS, non-ribosomal peptide synthetase cluster; T1PKS, type I PKS (Polyketide synthase); NRPS-like, NRPS-like fragment; T3PKS, type III PKS; T2PKS, type II PKS; PKS-like, other types of PKS cluster; LAP, linear azol(in)e-containing peptides; HglE-KS, heterocyst glycolipid synthase-like PKS; CDPS, cyanobactins like patellamide; Amglyccycl, aminoglycoside/aminocyclitol cluster; Blactam, β-lactam cluster; TfuA-related, TfuA-related RiPPs; Hserlactone, homoserine lactone cluster; Fused, pheganomycin-style protein ligase-containing cluster; Other: cluster containing a secondary metabolite-related protein that does not fit into any other category.

The set of *Streptomyces* strains analyzed carry 41 different types of BGCs out of 52 types defined by antiSMASH. The diversity in each genome goes between 10 and 26 types of BGCs (average and median=18). *Streptomyces lydicus* WYEC 108 was the strain that displayed the higher diversity, and *Streptomyces koyangensis* VK-A60T the lowest one. NRPS, terpene, and siderophore clusters were present in the 121 genomes (Figure 5; Supplementary Figure S4); although T1PKS and bacteriocin clusters were present in most of the strains, they were not found in *Streptomyces exfoliatus* A1013Y and *Streptomyces xiamenensis* 318, respectively. Furthermore, the ribosomally synthesized and post-translationally modified peptide (RiPP) clusters bottromycin and cyanobactin were only found in *Streptomyces scabiei* 87.22, and *S. lydicus* A02, respectively.

Although, no obvious relationship between the source of strains and their BGCs were found, there is a slight association between the frequency of BGC types and the genetic proximity (Supplementary Figure S4). For example, the clade of *S. hygroscopicus* displays similar frequency of NRPS, terpene, T1PKS, and siderophore; only the variety *limoneus* KCTC 1717 exhibited more bacteriocins in comparison to the varieties *jinggangensis* 5008 and the engineered *jinggangensis* TL01. In the case of *S. lividans* TK24 and *S. coelicolor* A3(2), both display a similar frequency of BGCs; yet, only *S. lividans* contains more terpenes in its genome. An interesting comparison is between *Streptomyces* sp. CNQ-509 and *Streptomyces* sp. WAC 06738; both strains are in the same clade but come from different isolation sources, marine and soil respectively, and mainly differ in the number of NRPS and T1PKS in their genomes.

**Figure 6:**
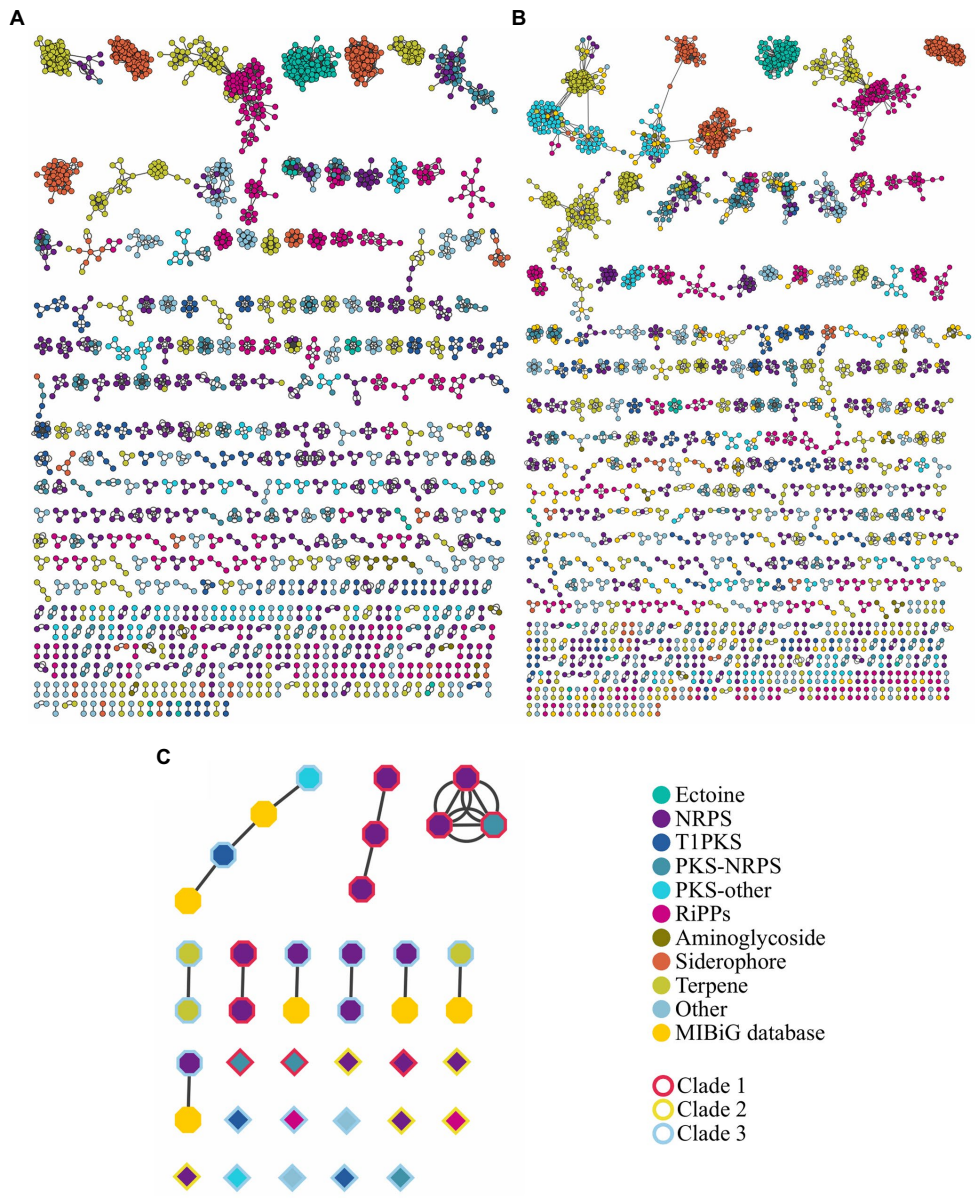
Sequence similarity network of BGCs **(A)** without and **(B)** with the information of the Minimum Information about a Biosynthetic Gene Cluster (MIBiG) database. **(C)** Sequence similarity network of prioritized BGCs; borders of figures represent the color code of the phylogenetic tree in Supplementary Figure S4. Analysis was made using BiG-SCAPE at cutoff=0.3. NRPS includes NRPS-like; PKS-other includes T2PKS, T3PKS, PKS-like and hglE-KS; RiPPs include bacteriocin, lanthipeptide, linear azol(in)e-containing peptides (LAP), lassopeptide, thiopeptide, and TfuA-related. Others include hybrid clusters different from PKS-NRPS.

The high BGCs variability in the genus was demonstrated with the cluster region comparison using BiG-SCAPE. This bioinformatic tool estimates distances between BGCs through the combination of the Jaccard index to determine the similarity of protein domains in the BGCs, the adjacency index that indicates the adjacent domains shared between BGCs, and the domain sequence similarity index, which calculate the sequence identity along with the domain copy number differences (Navarro-Muñoz et al., 2020). The network created by BiG-SCAPE using a cutoff of 0.3 – to identify interactions between BGCs producing similar compounds – displayed 2,359 nodes, and 12,969 edges (Figure 6A). A further comparison showed that 838 regions out of the 3,750 identified by antiSMASH are similar or have been already reported in the MIBiG database (Figure 6B; Supplementary File S4). Terpene, NRPS, siderophore, and ectoine are the clusters with the largest network similarity, whereas 1,204 cluster regions are unique within the analyzed genomes (Supplementary File S5).

To prioritize the search for antibiotics, ARTS uses BGC prediction from antiSMASH and displays the presence of self-resistance enzymes co-localized with BGC. In all 121 genomes analyzed, only 593 self-resistance genes were identified, distributed in 480 cluster regions out of the 3,750 regions identified by antiSMASH. On average, we identified five self-resistance genes in a genome; the maximum amount of self-resistance genes found in a genome was 12, in *Streptomyces alfalfae* ACCC40021. *Streptomyces globisporus* TFH56 was the unique strain without a self-resistance enzyme identified in its genome. Nevertheless, this strain can inhibit the growth of *Botrytis cinerea*, a gray mold pathogen that grows in tomato flowers (Cho and Kwak, 2019). Furthermore, we observed that NRPS and T1PKS are more frequently co-localized with self-resistance genes in comparison to other BGCs (Figure 5).

Another feature, considered in the prioritization of BGCs as possible producers of antibiotics, is the identification of core genes (defined by ARTS) within a biosynthetic cluster. Thus, 3,040 core genes were found in all genomes distributed in 1,490 regions. NRPS, terpene, and T1PKS offer the highest number of identified core genes (Figure 5). Additionally, core genes along with self-resistance genes were found in only 242 regions, being NRPS, T1PKS, and T2PKS the BGCs more frequently co-localized with both types of genes (Figure 5). Since some antibiotics target core genes, the producing bacteria tend to duplicate the gene and produce a homolog to avoid suicide. In this way, the presence of duplicated core genes in the BGC could lead to the prediction of the mode of action of the encoded antibiotic (Mungan et al., 2020). Applying a stricter filter to predict the antibiotic with its correspondent target, we only found 33 regions (distributed in 31 genomes) co-localized with self-resistance genes and core genes with evidence of duplication and HGT (Table 1); most of these clusters were NRPS (Figure 5).

**Table 1:** Prioritized BGCs for their putative antibiotic biosynthesis production.

Organism name	# Cluster	Core gene description	BGC type	Resistance model	MIBiG report^1^
*Streptomyces avermitilis* MA-4680	18	Proteasome, beta subunit	T2PKS, T1PKS	Proteasome subunit	Spore pigment. Similarity with curamycin from *S. cyaneus*
	19	GAPDH type I	Terpene	GAPDH_C	Pentalenolactone
*Streptomyces bingchenggensis* BCW-1	10	GAPDH type I	NRPS, furan, T1PKS, hglE-KS	GAPDH_C	Not reported
	20	Proteasome, beta subunit	T1PKS, NRPS	Proteasome subunit	Not reported
*Streptomyces violaceusniger* Tu 4113	21	GAPDH type I	NRPS-like	GAPDH_C	Not reported
*Streptomyces* sp. Sv. ACTE SirexAA-E	1	GAPDH type I	NRPS	GAPDH_C	Not reported
*Streptomyces cattleya* NRRL 8057	2_13	GAPDH type I	NRPS-like	GAPDH_C	Not reported
*Streptomyces hygroscopicus jinggangensis* 5008	15	GAPDH type I	NRPS	GAPDH_C	Not reported
*Streptomyces hygroscopicus jinggangensis* TL01	15	GAPDH type I	NRPS	GAPDH_C	Not reported
*Streptomyces fulvissimus* DSM 40593	2	GAPDH type I	NRPS	GAPDH_C	Not reported
*Streptomyces collinus* Tu 365	26	GAPDH type I	Terpene	GAPDH_C	Not reported
*Streptomyces cyaneogriseus noncyanogenus* NMWT 1	24	GAPDH type I	Terpene	GAPDH_C	Not reported
*Streptomyces* sp. CdTB01	28	GAPDH type I	T1PKS	GAPDH_C	Not reported
*Streptomyces* sp. SAT1	24	GAPDH type I	Lanthipeptide	GAPDH_C	Not reported
*Streptomyces lydicus* 103	22	GAPDH type I	NRPS, other, T3PKS	GAPDH_C	Not reported
*Streptomyces puniciscabiei* TW1S1	1	DNA polymerase III, beta subunit	Terpene, T1PKS	DNA polymerase III, beta subunit	Not reported
*Streptomyces autolyticus* CGMCC0516	5	GAPDH type I	NRPS-like	GAPDH_C	Not reported
*Streptomyces lydicus* GS93/23	16	GAPDH type I	T3PKS, NRPS, other	GAPDH_C	Not reported
*Streptomyces niveus* SCSIO 3406	24	GAPDH type I	NRPS	GAPDH_C	Not reported
*Streptomyces hygroscopicus* XM201	5	GAPDH type I	NRPS-like	GAPDH_C	Not reported
*Streptomyces* sp. MOE7	15	GAPDH type I	Other, T3PKS, NRPS	GAPDH_C	Not reported
*Streptomyces lavendulae lavendulae* CCM 3239	21	GAPDH type I	Thiopeptide, LAP	GAPDH_C	Not reported
*Streptomyces* sp. M56	43	GAPDH type I	NRPS-like	GAPDH_C	Not reported
*Streptomyces* sp. P3	31	GAPDH type I	NRPS	GAPDH_C	Similarity with scabichelin from *S. scabiei*
*Streptomyces lunaelactis* MM109	27	GAPDH type I	NRPS-like	GAPDH_C/Metallo-β-lactamase	Not reported
*Streptomyces nigra* 452	20	GAPDH type I	T3PKS	GAPDH_C	Not reported
*Streptomyces* sp. ZFG47	23	GAPDH type I	T2PKS	GAPDH_C	Similarity with curamycin from *S. cyaneus*
*Streptomyces* sp. 11-1-2	4	GAPDH type I	NRPS-like	GAPDH_C	Not reported
*Streptomyces* sp. WAC 01438	18	GAPDH type I	T3PKS, NRPS, T2PKS	GAPDH_C/GAPDH_C	Similarity with spore pigment from *S. collinus*
*Streptomyces* sp. WAC 01529	1	GAPDH type I	Lassopeptide, NRPS, terpene	GAPDH_C	Not reported
*Streptomyces* sp. GGCR-6	4	GAPDH type I	T1PKS	Carboxyl transferase domain/GAPDH_C	Not reported
*Streptomyces* sp. MK-45	4	GAPDH type I	NRPS	GAPDH_C	Similarity with isocomplestatin from *S. lavendulae*
*Streptomyces* sp. endophyte N2	3	GAPDH type I	NRPS-like, T1PKS	GAPDH_C	Not reported

GAPDH, glyceraldehyde 3-phosphate dehydrogenase; and GAPDH_C, glyceraldehyde 3-phosphate dehydrogenase, C-terminal domain.

1 Similarity found using BiG-SCAPE including the MIBiG database at cutoff 0.3.

In these 33 regions (Table 1), core genes were also classified as self-resistance genes; the diversity of these genes was low, presenting only three functions: glyceraldehyde-3-phosphate dehydrogenase (GAPDH) type I, proteasome, and DNA polymerase III β-subunit. Two self-resistance genes within the same region were found in only three genomes. In a NRPS-like cluster of *Streptomyces lunaelactis* MM109 the resistance targets were found in the C-terminal domain of GAPDH (GAPDH_C) and a metallo-β-lactamase, whereas in *Streptomyces* sp. WAC 01438 the T3PKS/NRPS/T2PKS cluster displayed two GAPDH_C as self-resistance genes, and *Streptomyces* sp. GGCR-6 presented a carboxyl transferase domain and GAPDH_C as resistance targets in a T1PKS cluster.

Using the approach of BGC prioritization, we identified BGCs with all elements needed to biosynthesize antibiotic molecules with a predicted mode of action. Some of the prioritized BGC display similarity with another prioritized cluster from a genetically related *Streptomyces* (Figure 6C), i.e., region 22 of *S. lydicus* 103 is similar to the region 16 of *S. lydicus* GS93 and the region 15 of *Streptomyces* sp. MOE7. Also, the region 5 of *S. hygroscopicus* XM201, the region 21 of *Streptomyces violaceusniger* Tu 4113 and the region 4 of *Streptomyces* sp. 11-1-2 are similar from each other. Likewise, the region 5 of *S. autolyticus* CGMCC0516 and the region 43 *Streptomyces* sp. M56 share sequence similarity. Intriguingly, the region 26 of *Streptomyces collinus* Tu 365 and the region 24 of *S. cyaneogriseus noncyanogenus* NMWT 1 are similar but both strains are not closely genetically related.

Of our prioritized BGCs only the region 19 of *Streptomyces avermitilis* MA-4680 is already described as the biosynthetic pathway of the antibiotic pentalenolactone, the region 18 also of *S. avermitilis* MA-4680 is reported in the MIBiG database (Kautsar et al., 2020) as a spore pigment cluster (although a bioactivity assay is not reported). Other four regions, along with the region 18 of *S. avermitilis* MA-4680, have similarity with a reported cluster in the MIBiG database (Table 1). Thus, we were able to perform a high throughput antibiotic screening using the bioinformatic tool ARTS 2.0, identifying interesting clusters that could be experimentally tested.

## Discussion

A pan-genome, defined as the entire set of non-orthologous genes in a specified group of strains (Tettelin et al., 2008), may reveal gene clusters of special interest as those related with specific niches or involved in the production of bioactive compounds (Medini et al., 2005). This study aims to determine the pan-genome or supra-genome of the genus *Streptomyces*. From all genomes available in NCBI, only 121 complete genomes with high quality assemblies were selected for the analysis. In addition, two approaches to compute the gene families or clusters were explored: the sequence similarity, using the software Roary (Page et al., 2015), and, based on the presence of common protein domains, using the R package Micropan (Snipen and Liland, 2015).

The analysis with Roary exposes a pan-genome size of 145,462 gene families; 94.7% of them corresponds to cloud genes. This finding is consistent with the study of Xu et al. (2019) who uncovered 123,302 clusters in 87 genomes of *Streptomyces* derived from marine ecosystems; the authors used genomes with completeness above 95% and employed an identity of 50% for clustering, which can impact the pan-genome size. In another study, 39,893 gene families across the genus were determined in a study using a similar number of *Streptomyces* strains (122; McDonald and Currie, 2017). To generate gene families, the authors used Proteinortho v2 (Lechner et al., 2011) with default parameters; this tool uses a low value of percent identity (25%) as a threshold, which might be the cause of any difference with our results. Besides, many of the genomes the authors used are fragmented, which can introduce errors in pan-genome calculations (Tonkin-Hill et al., 2020). A recent paper reported a pan-genome size of 106,000 genes and 1,018 core genes by using 125 complete *Streptomyces* genomes and a percent similarity threshold of 40% in BLASTp (Lorenzi et al., 2021); this might explain the differences with the present study, although, the core genes number is quite similar to the soft-core genes that we calculated. What is remarkable in these two approaches is the similar value of gamma (γ) in the mathematical fit of the genome size (0.62 compared to the 0.6 obtained in this work). As the identity of the strains used in both studies also differs, this similar gamma (γ) value states that the quality of the genomes is the most important feature to obtain reliable results and predictions. Using the pan-genome size estimation performed by BinomixEstimate (273,372 clusters) and the value of gamma (γ), we estimate that around 284 genomes are necessary to determine the complete reservoir of genes in the genus *Streptomyces*. The number of strains used may also cause a bias in the analysis. Because of this, related pan-genomic studies in *Streptomyces* determined significantly less clusters than those found in this investigation (Kim et al., 2015; Tian et al., 2016; Wu et al., 2017b; Jackson et al., 2018; Almeida et al., 2019). We also observed that the core genome size is higher in those studies that include few genomes, obtaining values greater than 2,000 core gene families (Zhou et al., 2012; Kim et al., 2015; Tian et al., 2016); this value tends to decrease as more genomes are added.

To our knowledge, no previous characterization of the pan-genome of the genus *Streptomyces* has been performed based on protein domains. This is an alternative approach that is robust against errors in predicting of protein coding genes, which reduces the variation in annotation between genomes (Snipen and Ussery, 2013). Surprisingly, the number of clusters reduces dramatically compared to the calculations carried out by Roary, although, the number of core genes remains similar. It is possible to argue that many proteins without domain annotations are discarded in the Micropan analysis and that is the case of cloud proteins, which are poorly characterized because they are less frequently found and therefore less studied. This inference is supported by the fact that COG annotations of core genomes of both methods are quite consistent, while the proportion of COG categories in the shell and cloud genomes differs markedly. Conversely, the threshold used to consider a protein as belonging to the same cluster could be high if we consider that we are characterizing a genus with enormous genetic variety. Nevertheless, some proteins can have similar function and therefore similar domains in their structures; as a result, their protein sequence identity can still be low to be clustered in the same group even if we reduce the threshold. This idea is strengthened by an additional analysis with the pipeline BPGA (Chaudhari et al., 2016) using a 50% of identity. Here, 662 core gene families were obtained; this outcome is very similar to the sizes reported by the methods used in the present study. Moreover, we also found a higher number of unique genes (48,315, data not shown), which were less than those found with Roary, where the threshold was 70%. The gap between the number of clusters, from Roary and Micropan, could be attributed to false predicted “genes,” which do not align correctly to other clusters producing an increase in the number of unique genes or singletons (Snipen and Ussery, 2013). Further, overestimation of cloud genes has been previously reported when using Roary and other methods, not based on protein domains, to estimate pan-genome sizes (Tonkin-Hill et al., 2020).

Regarding the diversity of the *Streptomyces* spp., the genomic fluidity and the Jaccard distance were determined for the pan-genomes produced by Roary and Micropan. These results seem to be consistent with an open pan-genome with a high and diverse gene content. Overall, fluidity values tend to be low for species and increase as genetic distance arises, e.g., for *Emiliania huxleyi* (Read et al., 2013) and for *Burkholderia pseudomallei* (Spring-Pearson et al., 2015) this value has been estimated in 0.1 and 0.17, respectively; a notable exception is *Cronobacter sakazakii* which has a fluidity of 0.875, which indicates a large accessory genome pool of this specie (Lee and Andam, 2019). At the level of genus Kislyuk et al. (2011) calculated a fluidity value around 0.9 for the genus *Frankia*, which belongs to the phylum *Actinobacteria*. In a recent study, a value of 0.12 was obtained for *Streptomyces rimosus* (Park and Andam, 2019). We considered the fluidity value for *Streptomyces* spp. as a reasonable assessment strategy of the genus’ diversity; this value reveals the enormous diversity of strains exposed to different lifestyles and habitats, and therefore, prone to acquire genetic material through lateral transfer so as to obtain better adaptations to their environments; undoubtedly, this results in a wide range of the genome sizes and protein coding genes in streptomycetes (see Supplementary Figure S2). Consequently, some strains have almost the double of protein coding genes.

Micropan results are more difficult to compare because this methodology is less employed in pan-genomic studies, yet the fluidity obtained with this software is quite low compared with the one obtained with Roary. It may indicate that, in terms of functionality of the genomes, the dissimilarity diminishes around 20%; therefore, many clusters, which are separated when sequence similarity is used to form them, can have the same or similar function due to the presence of the same domains in its sequence.

Small RNAs play an important role in post-transcriptional control of messenger RNA expression and regulate diverse processes, e.g., carbon metabolism, iron homeostasis, RNA polymerase function, virulence, biofilm formation, oxidation, outer membrane perturbation, cellular accumulation of sugar-phosphates and plasmid replication (Richards and Vanderpool, 2011). Trans-encoded regulatory sRNAs are located at sites distinct from those of their target genes and they are typically encoded and enriched in the conserved IGRs of bacterial genomes (Tsai et al., 2015). Therefore, a precise determination of conservation of IGR is a crucial stage in small-RNAs studies as this is typically the first step in the computational identification of these important regulators in bacteria (Rossi et al., 2016; Fuli et al., 2017). Some software use this information to predict novel sRNAs in bacterial genomes such as RNAz (Gruber et al., 2010) and QRNA (Sridhar and Gunasekaran, 2013). Since little is known about the abundance and function of sRNA in Gram-positive bacteria like *Streptomyces* (Engel et al., 2020), an accurate determination of the conservation of IGRs and its dependency with phylogenetic distance is necessary for a proper estimation of regulatory RNAs encoding potential (Tsai et al., 2015). The current analysis shows that IGRs conservation is reduced at the level of genus and the conservation is still low in smaller groups, when strains are grouped according to the three clades obtained in the phylogenetic tree. However, these rarely conserved IGRs can harbor regulatory function since novel putative non-coding RNAs (nc-RNAs) were detected in these regions; the role of these putative ncRNAs is an interesting question because a high selection pressure must act to conserve these sequences in species with an enormous diversity such as streptomycetes indicating their participation in controlling multiple metabolic processes. As a first approach, we investigated the interaction of these molecules with other functional RNAs showing that numerous mRNAs with diverse annotations (Supplementary File S2) can interact with these predicted regulators. By other hand, we hypothesize that reducing the genetic distance among species will produce trustworthy alignments, which plays a key role in the RNAs structure prediction and will improve the bioinformatics predictions. This is reinforced by the fact that, when the analysis is made in more related strains, i.e., at the level of species, IGRs are well preserved. Therefore, the current analysis lays the foundations for further studies involving computational predictions of sRNAs and their regulatory mechanism in species with biotechnological application such as *S. clavuligerus*, *S. hygroscopicus*, *S. lydicus*, and *S. albus*.

A high confidence phylogenetic tree, using 633 markers, was obtained as a result of core genome determination in the pan-genome analysis. Overall, there is a strong resemblance with earlier phylogenomic analysis performed in *Streptomyces* by Martín-Sánchez et al. (2019) who used 93 complete *Streptomyces* genomes and 575 markers. McDonald and Currie (2017) also obtained similar results, though their analysis included fragmented genomes and the bootstrap values of some branches were less than 0.7, which is considered a low bootstrap support. In that study, the number of markers was inferior (94), and many genomes were fragmented. Thus, as it was already highlighted, our first and foremost priority would be to decide on high quality genomes for confident evolutionary analysis.

What is striking in our analysis is the correlation found during ANI determination for strains with values above 95%, and their position in the core genome tree. Together with the core genome tree, ANI calculations consider only the part of the genome, where alignments can be built (Richter et al., 2016). Global alignments of strains with ANI values above 95% support differences among genomes despite the high conservation in their core genes (Supplementary Figures S22–S31); hence, it should be noted that genomic analyses along with biochemical and physiological characterizations are still necessary for the correct taxonomic classification of microorganisms. By way of illustration, *S. coelicolor* A3(2) and *S. lividans* TK24 possess an ANI value that suggests they are the same species, or even the same strain, but their phenotypic behavior differs markedly. *Streptomyces lividans* TK24 produces small amounts of the antibiotics actinorhodin and undecylprodigiosin compared to *S. coelicolor* A3(2) (Rückert et al., 2015). With the advent of new and complete genomes, a deep analysis should be performed for a possible taxonomic re-classification of the species mentioned in Supplementary Figures S22–S31 (special attention must be paid to *S. hygroscopicus* XM201).

The genus *Streptomyces* is characterized for its metabolic capacity of producing a wide range of metabolites with high societal impact (Pham et al., 2019) and is still one of the most studied genera. *Streptomyces* is the genus with most entries in the MIBiG database by far (636 entries, search made on January 31, 2021), followed by *Aspergillus* and *Pseudomonas*.

Previous genome mining studies have been developed in the genus *Streptomyces*. Our findings correlate well with results previously reported by Belknap et al. (2020). Using antiSMASH 4.1 they predicted that NRPS, PKS1, terpenes, and lantipeptides were the most common BGCs, and *S. rhizosphaericus* NRRL B-24304 (not included in our study) carried the highest number of BGCs (*n*=83). The slight differences between our results and results reported in 2020 might be caused by improvements in BGC detection found in newer versions of antiSMASH (Blin et al., 2019), as well as the number of genomes analyzed and their quality.

In our study, the ribosomally synthesized and post-translationally modified peptide (RiPP) clusters bottromycin and cyanobactin were only found in *S. scabiei* 87.22, and *S. lydicus* A02, respectively. Surprisingly, as far as we know, there are no reports of cyanobactin expression in *Streptomyces* strains; cyanobactin clusters were previously identified in *S. lydicus* A02 and *S. venezuelae* genomes using the genome mining tool BAGEL3 (Poorinmohammad et al., 2019). Bottromycin, however, is already described in *S. scabies* DSM 41658 (Vior et al., 2020). In a recent study, where 1,110 genomes of *Streptomyces* strains were analyzed (including incomplete genomes), cyanobactin and bottromycin clusters were identified in seven and 17 genomes, respectively (Belknap et al., 2020), demonstrating that, despite the fact that these BGCs were rarely found in the set of genomes we analyzed, it does not mean that other BGCs could not be present in other *Streptomyces* strains out of the scope of the present study.

Cluster similarity analysis demonstrated that terpenes are also highly similar in the genus as previously reported (Martín-Sánchez et al., 2019). Siderophore and ectoine are also highly similar probably due to their primary role in iron acquisition and stress protectant, respectively (Jones et al., 2019; Richter et al., 2019). Intriguingly, one third of the predicted cluster regions did not display similarity with other predicted or reported region, and only one fifth of the prioritized antibiotics are similar to a reported cluster, demonstrating the capacity of the genus to produce diverse compounds.

It is well established that BGCs of known antibiotics produced by *Streptomyces* are co-localized with self-resistance enzymes, e.g., streptomycin and cephamycin C produced by *S. griseus* and *S. clavuligerus*, respectively (Supplementary File S3). Regions containing both clusters were successfully found by ARTS along with other 478 regions with co-localized self-resistance enzymes. The challenge now is the creation of strategies to prioritize the identification of BGCs with novel antibiotic activity within the increasing genomic data. As an approach to rationalize the seek for antibiotics, Culp et al. (2020) proposed that identifying BGCs with low similarity and lacking known resistance determinants could lead to the detection of antibiotics with novel mechanisms of action. Following this strategy, they identified two glycopeptide bacteriostatics with an unknown mechanism of action (Culp et al., 2020). Thus, the identification of BGCs nearby self-resistance enzymes along with duplicated core genes with predicted HGT, seems to be a promissory approach to identify BGCs that potentially produce new antibiotics with a predicted mode of action; this approach is currently used in the quest for new antibiotic clusters (Yan et al., 2020) and led to the discovery of thiolactomycin in *Salinispora pacifica* (Tang et al., 2015). ARTS is the first tool to incorporate these parameters that could derive more confident predictions (Tran et al., 2019); it is a powerful tool and user friendly for a high throughput identification of BGCs for potential antibiotic biosynthesis. Despite its ease of use and how informative is, only few studies have incorporated ARTS in their methodologies. In this regard, we call the attention to the analysis of marine myxobacterial strains, which revealed these strains contain a high number of self-resistance genes, e.g., *E. salina* DSM 15201 contains 13 self-resistance genes (Moghaddam et al., 2018). We strongly recommend that bioinformatics tools such as ARTS should be incorporated in further studies aimed at seeking for new antibiotics.

Using ARTS, we prioritized the search of cluster regions with a predicted mode of action. As part of our predictions, we successfully identified the pentalenolactone cluster, which targets indeed the glyceraldehyde-3-phosphate dehydrogenase (Cane and Sohng, 1989). Some of the prioritized regions are co-localized with more than one self-resistance gene that could increase the probability of an antibiotic activity. The most promising of the prioritized regions could be the region 23 of *Streptomyces* sp. ZFG47 and the region 18 of *S. avermitilis* MA-4680 since both displayed a similarity with the antibiotic curamycin from *Streptomyces cyaneus* (Figure 6C; Table 1).

Parameters like duplication and HGT of core genes should be used carefully if the purpose is the identification of any type of antibiotics, since filters with these parameters exclude the high number of clusters settled nearby core and self-resistance genes, which, along with biosynthetic clusters of antibiotics already known, might be potentially used for metabolic reengineering strategies to produce new antibiotic scaffolds. It is worth stressing that the metabolic potential of the genus *Streptomyces* goes beyond antibiotics, and with every new discovered species, we may possibly be amazed by their metabolic complexity and richness. Without a doubt, this genus is and apparently will continue to be one of the most fascinating to be studied.

## Data Availability Statement

The datasets analyzed for this study can be found in the Reference Sequence (RefSeq) database (https://ftp.ncbi.nlm.nih.gov/genomes/refseq/bacteria/). The accession number for the genomes included can be found in the Supplementary File S1.

## Author Contributions

CC-M and RR-E designed the study. CC-M and MM-R collected the data, performed all bioinformatics analyses, and drafted the manuscript. RR-E supervised the research work, interpreted the results, corrected and wrote the manuscript, and serve as corresponding author. All authors contributed to the article and approved the submitted version.

## Funding

This work was supported by a grant obtained from MINCIENCIAS – Colombia – Convocatoria 785 – 2017. Grant # 80740-595-2019 to CC-M.

## Conflict of Interest

The authors declare that the research was conducted in the absence of any commercial or financial relationships that could be construed as a potential conflict of interest.

## Publisher’s Note

All claims expressed in this article are solely those of the authors and do not necessarily represent those of their affiliated organizations, or those of the publisher, the editors and the reviewers. Any product that may be evaluated in this article, or claim that may be made by its manufacturer, is not guaranteed or endorsed by the publisher.
