# Supplementary material for: Pan-Genome of the Genus Streptomyces and Prioritization of Biosynthetic Gene Clusters With Potential to Produce Antibiotic Compounds
Source: Front Microbiol. 2021 Sep 28;12:677558. doi: 10.3389/fmicb.2021.677558 (PMC8510958; doi:10.3389/fmicb.2021.677558)
Supplement: Supplementary file 6 [file Presentation_1.pdf]

Supplementary material

Pan-genome of the genus *Streptomyces* and prioritization of biosynthetic gene clusters with potential to produce antibiotic compounds

Carlos Caicedo-Montoya[1†], Monserrat Manzo-Ruiz[2†], Rigoberto Ríos-Estepa[1*]

[1]Grupo de Bioprocesos, Departamento de Ingeniería Química, Universidad de Antioquia, Medellín, Colombia

[2]Instituto de Investigaciones Biomédicas, Departamento de Biología Molecular y Biotecnología, Universidad Nacional Autónoma de México, Ciudad de México, México

*** Correspondence:**
Rigoberto Ríos-Estepa
rigoberto.rios@udea.edu.co

†These authors have contributed equally to this work

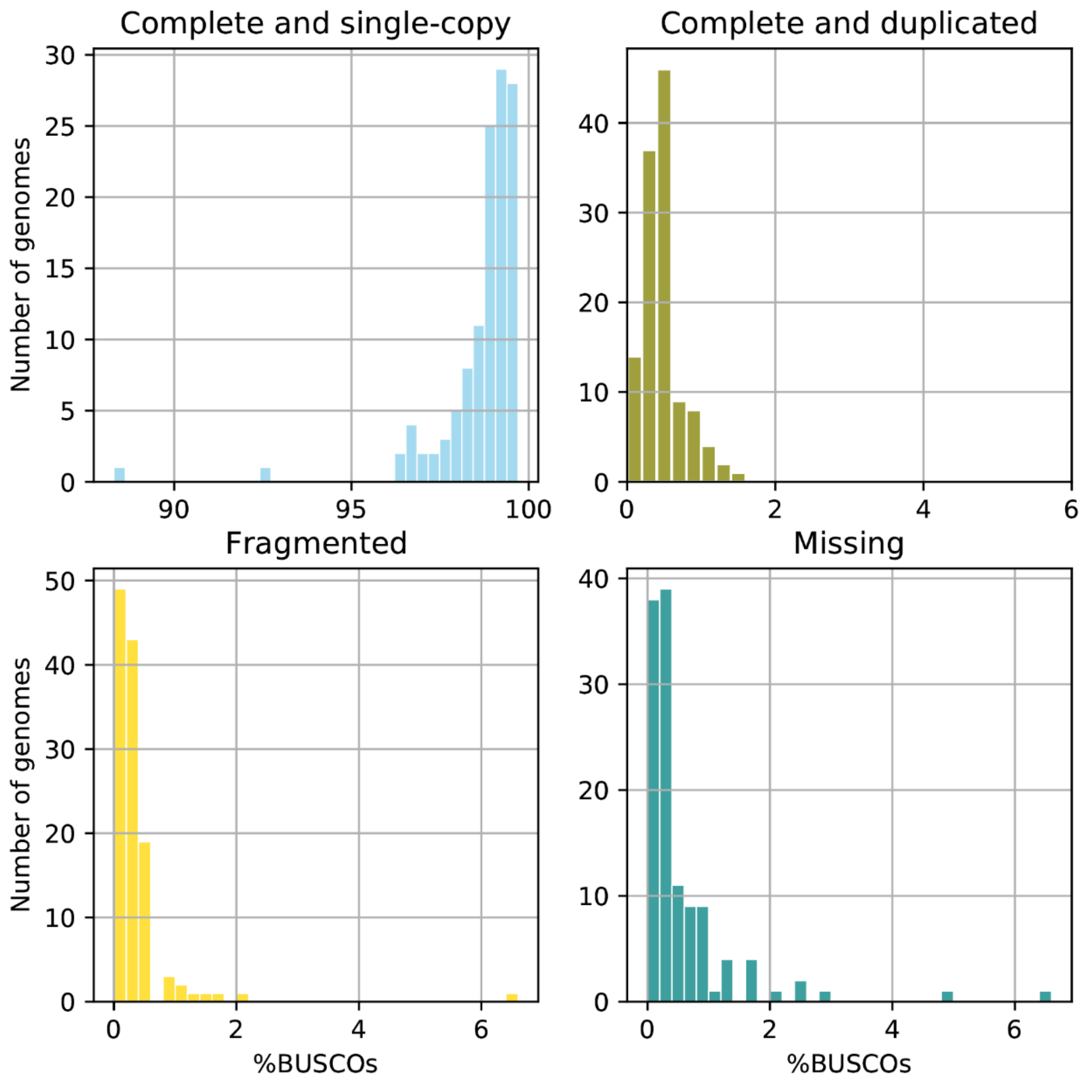

Supplementary Figure S1. Genome quality evaluation through Benchmarking Universal Single-copy Orthologs (BUSCOs).

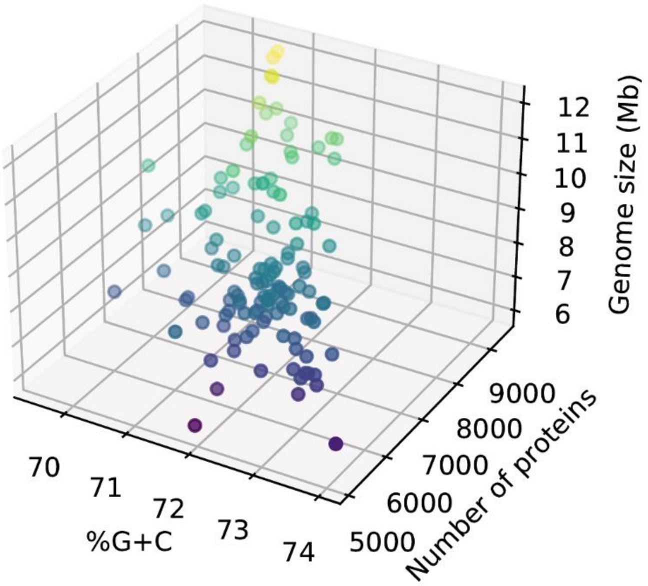

Supplementary Figure S2. Three-dimensional plot of genome size, number of proteins and percentage G+C of the 121 *Streptomyces* strains analyzed.

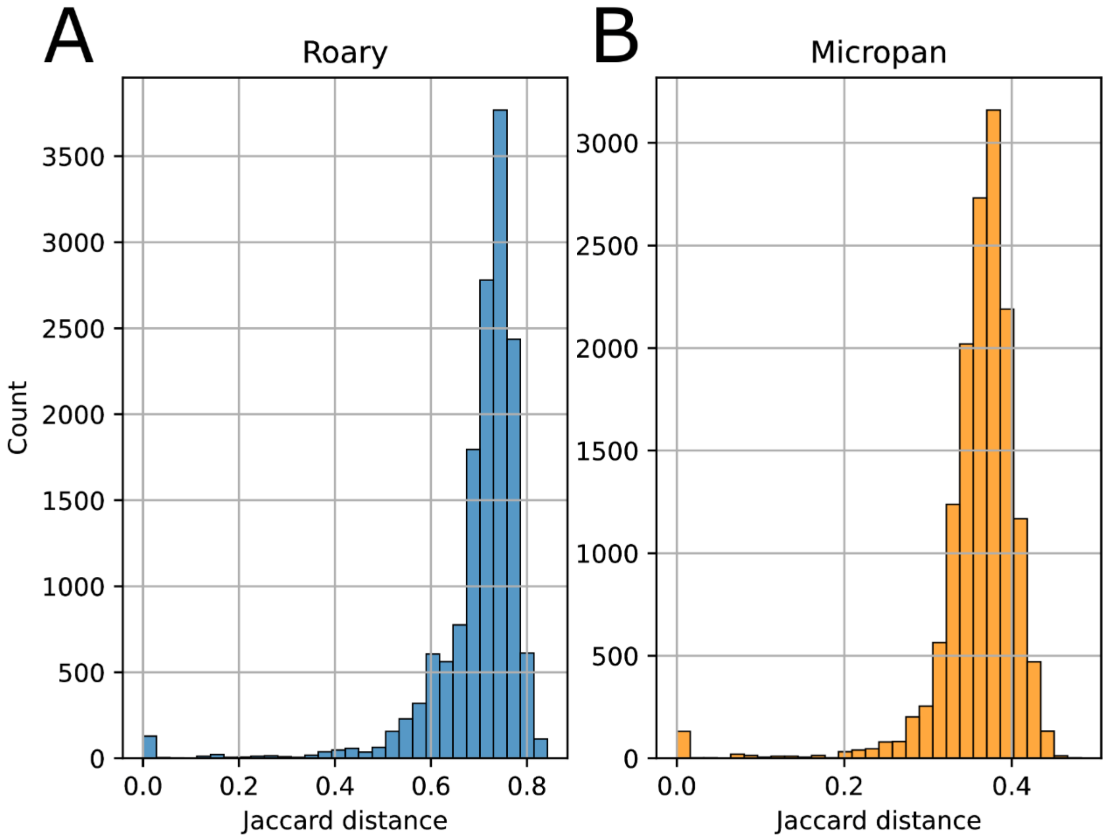

Supplementary Figure S3. Distribution of Jaccard distance for the genomes of 121 *Streptomyces* when presence or absence of a gene family in a genome is determined by (**A**) Roary and (**B**) Micropan. The Jaccard distance was evaluated using an approach of comparing all-against-all genomes. For two set of gene clusters belonging to a genome A and B, respectively, the Jaccard distance is defined as follow: $J(A,B) = 1 - \frac{|A \cap B|}{|A \cup B|}$

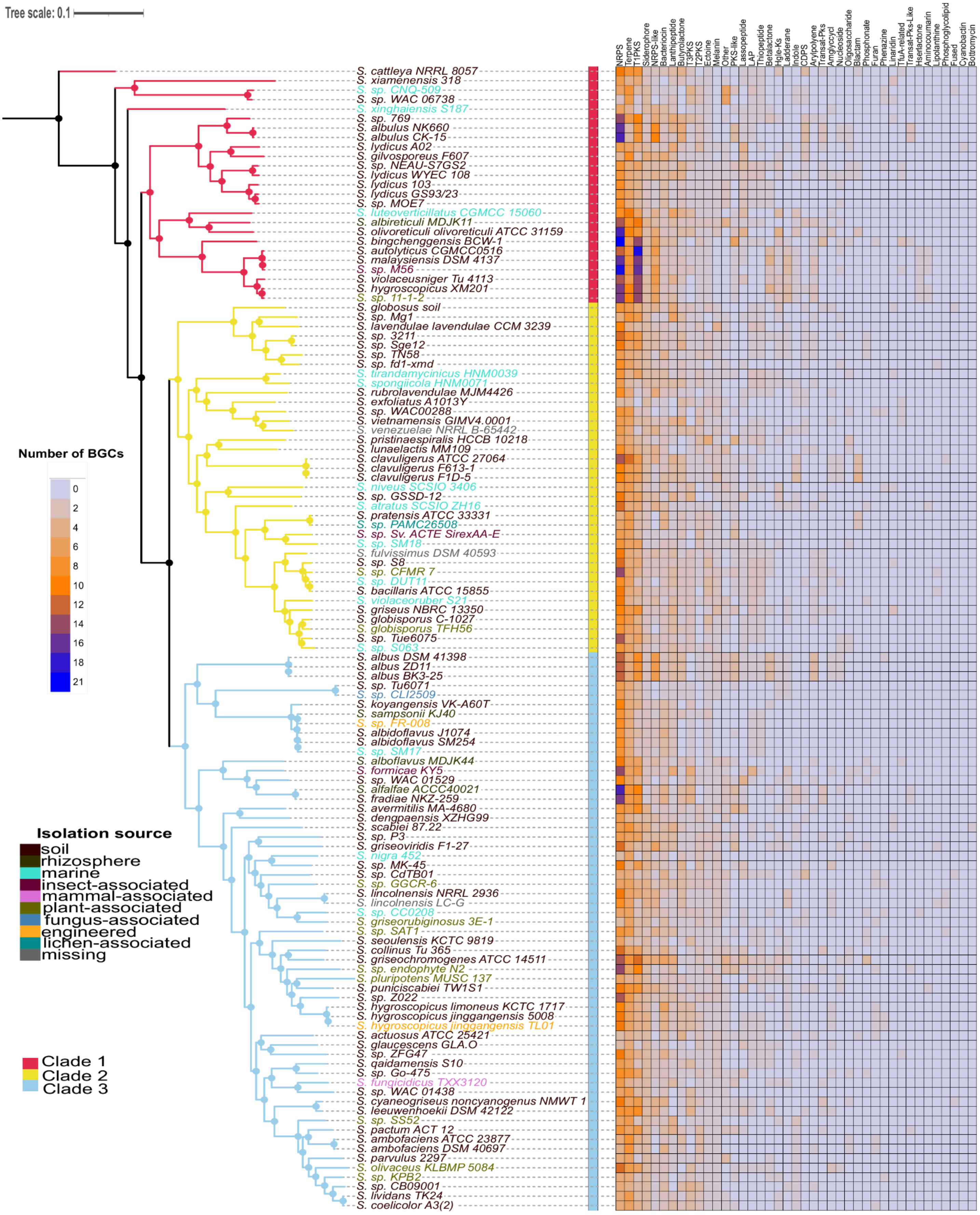

Supplementary Figure S4. Distribution of Biosynthetic Gene Clusters (BGCs) in the 121 *Streptomyces* strains analyzed. *Streptomyces* strains are organized by the core-genome phylogenetic tree and highlighted according to their isolation source. *Streptomyces* strains are segmented in 3 main clades. In red: clade1, yellow: clade 2 and blue: clade 3.

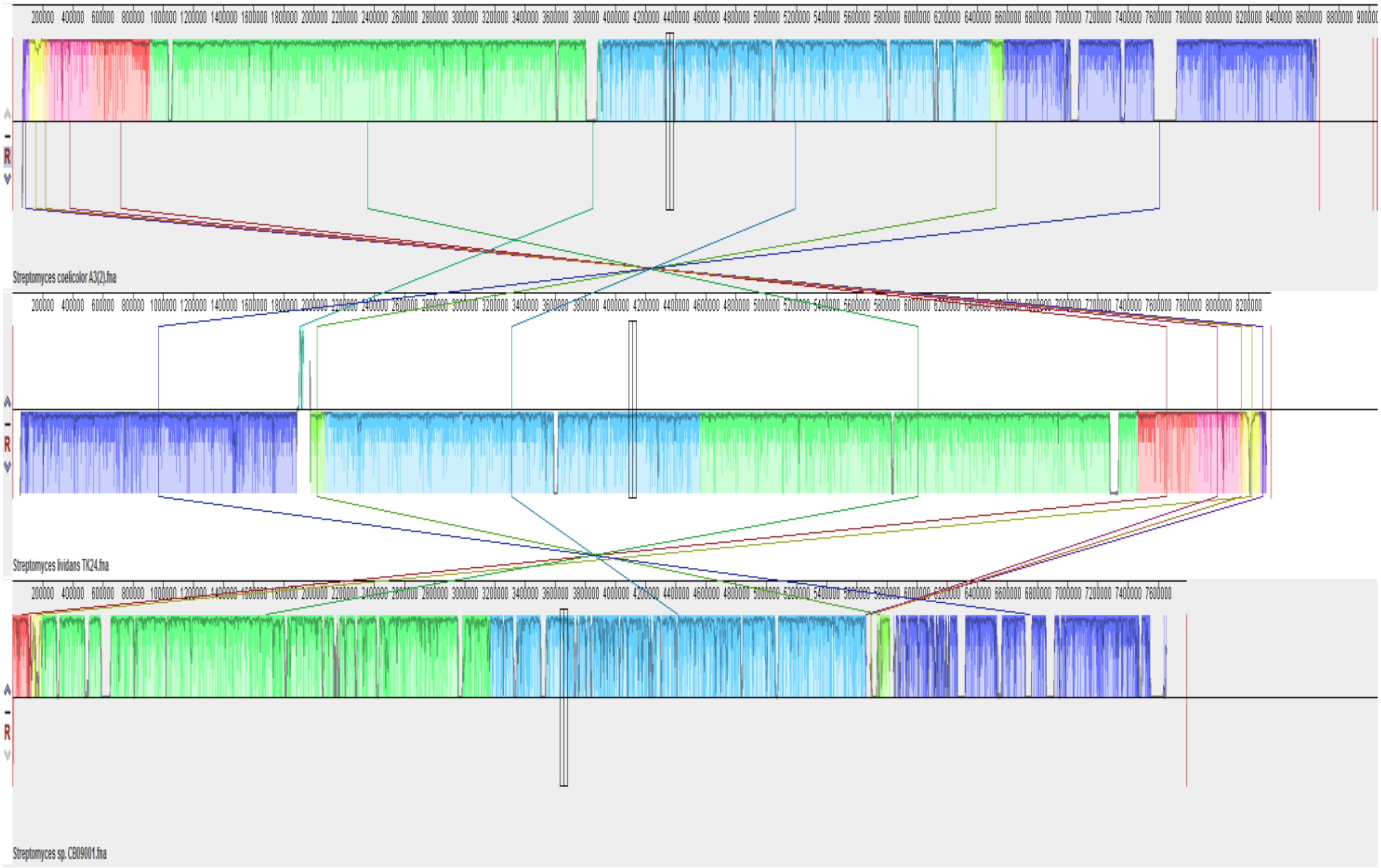

Supplementary Figure S5. Multiple genome alignment performed with the progressiveMauve algorithm for *S. coelicolor* A3(2), *S. lividans* TK24 and *Streptomyces* sp. CB0900.

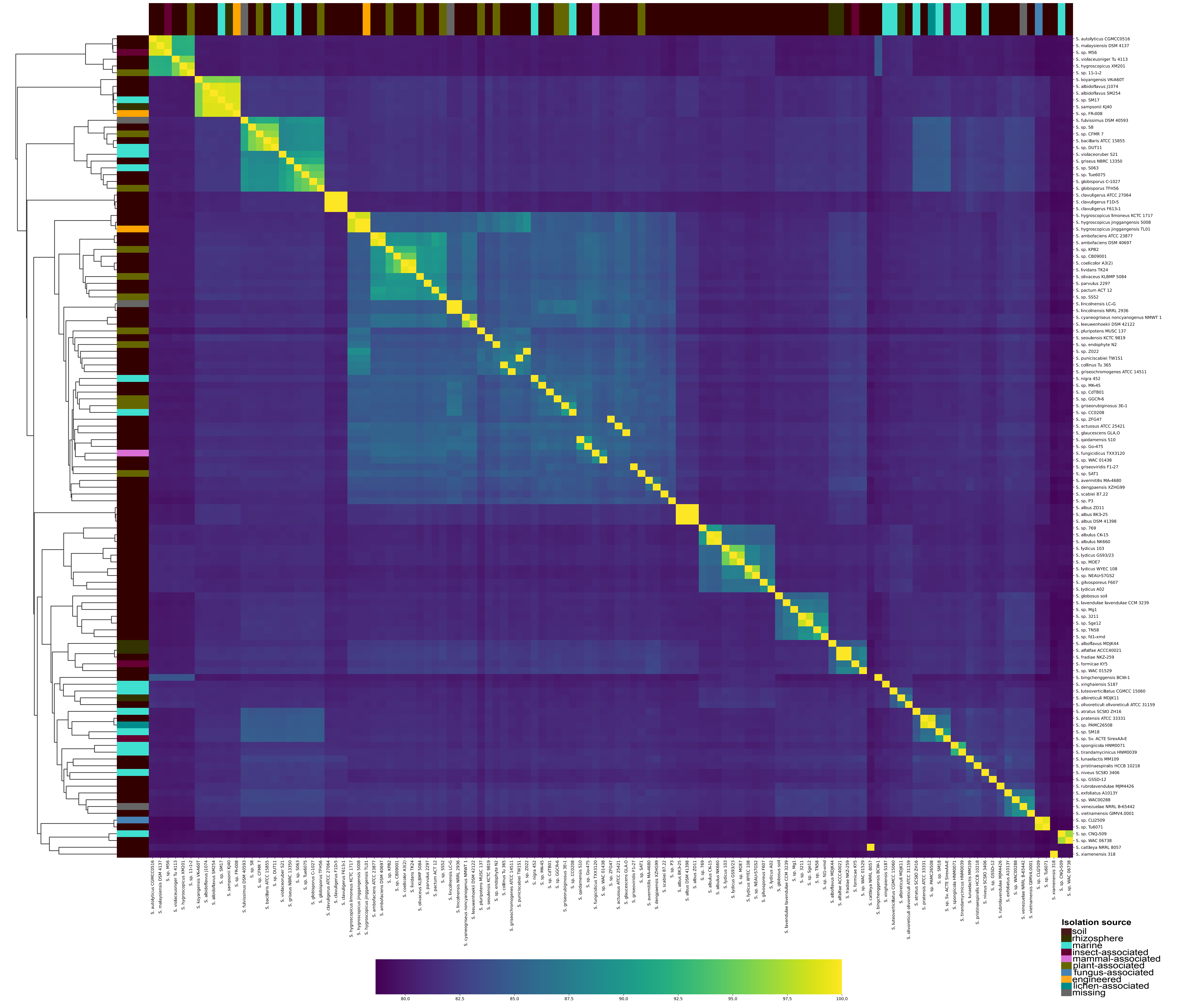
S. autolyticus CGMCC0516
S. malaysiensis DSM 4137
S. sp. M56
S. violaceusniger Tu 4113
S. hygroscopicus XM201
S. sp. 11-1-2
S. koyangensis VK-A60T
S. albidoflavus J1074
S. albidoflavus SM254
S. sp. SM17
S. sampsonii KJ40
S. sp. FR-008
S. fulvissimus DSM 40593
S. sp. S8
S. sp. CFMR 7
S. bacillaris ATCC 15855
S. sp. DUT11
S. violaceoruber S21
S. griseus NBRC 13350
S. sp. S063
S. sp. Tue6075
S. globisporus C-1027
S. globisporus TFH56
S. clavuligerus ATCC 27064
S. clavuligerus F1D-5
S. clavuligerus F613-1
S. hygroscopicus limoneus KCTC 1717
S. hygroscopicus jinggangensis 5008
S. hygroscopicus jinggangensis TL01
S. ambofaciens ATCC 23877
S. ambofaciens DSM 40697
S. sp. KPB2
S. sp. CB09001
S. coelicolor A3(2)
S. lividans TK24
S. olivaceus KLBMP 5084
S. parvulus 2297
S. pactum ACT 12
S. sp. SS52
S. lincolnensis LC-G
S. lincolnensis NRRL 2936
S. cyaneogriseus noncyanogenus NMWT 1
S. leeuwenhoekii DSM 42122
S. pluripotens MUSC 137
S. seoulensis KCTC 9819
S. sp. endophyte N2
S. sp. Z022
S. puniciscabiei TW1S1
S. collinus Tu 365
S. griseochromogenes ATCC 14511
S. nigra 452
S. sp. MK-45
S. sp. CdTB01
S. sp. GGCR-6
S. griseorubiginosus 3E-1
S. sp. CC0208
S. sp. ZFG47
S. actuosus ATCC 25421
S. glaucescens GLA.O
S. qaidamensis S10
S. sp. Go-475
S. fungicidicus TXX3120
S. sp. WAC 01438
S. griseoviridis F1-27
S. sp. SAT1
S. avermitilis MA-4680
S. dengpaensis XZHG99
S. scabiei 87.22
S. sp. P3
S. albus ZD11
S. albus BK3-25
S. albus DSM 41398
S. sp. 769
S. albulus CK-15
S. albulus NK660
S. lydicus 103
S. lydicus GS93/23
S. sp. MOE7
S. lydicus WYEC 108
S. sp. NEAU-S7GS2
S. gilvosporeus F607
S. lydicus A02
S. globosus soil
S. lavendulae lavendulae CCM 3239
S. sp. Mg1
S. sp. 3211
S. sp. Sge12
S. sp. TN58
S. sp. fd1-xmd
S. alboflavus MDJK44
S. alfalfae ACCC40021
S. fradiae NKZ-259
S. formicae KY5
S. sp. WAC 01529
S. bingchenggensis BCW-1
S. xinghaiensis S187
S. luteoverticillatus CGMCC 15060
S. albireticuli MDJK11
S. olivoreticuli olivoreticuli ATCC 31159
S. atratus SCSIO ZH16
S. pratensis ATCC 33331
S. sp. PAMC26508
S. sp. SM18
S. sp. Sv. ACTE SirexAA-E
S. spongiicola HNM0071
S. tirandamycinicus HNM0039
S. lunaelactis MM109
S. pristinaespiralis HCCB 10218
S. niveus SCSIO 3406
S. sp. GSSD-12
S. rubrolavendulae MJM4426
S. exfoliatus A1013Y
S. sp. WAC00288
S. venezuelae NRRL B-65442
S. vietnamensis GIMV4.0001
S. sp. CLI2509
S. sp. Tu6071
S. sp. CNQ-509
S. sp. WAC 06738
S. cattleya NRRL 8057
S. xiamenensis 318
80.0
82.5
85.0
87.5
90.0
92.5
95.0
97.5
100.0
Isolation source
soil
rhizosphere
marine
insect-associated
mammal-associated
plant-associated
fungus-associated
engineered
lichen-associated
missing

Supplementary Figure S6. Heat map representing the average nucleotide identity (ANI) values of the compared strains. The colors above the heat map represent the isolation source of the organisms. The dendrogram was generated with the UPGMA method available in the library Seaborn of Python.

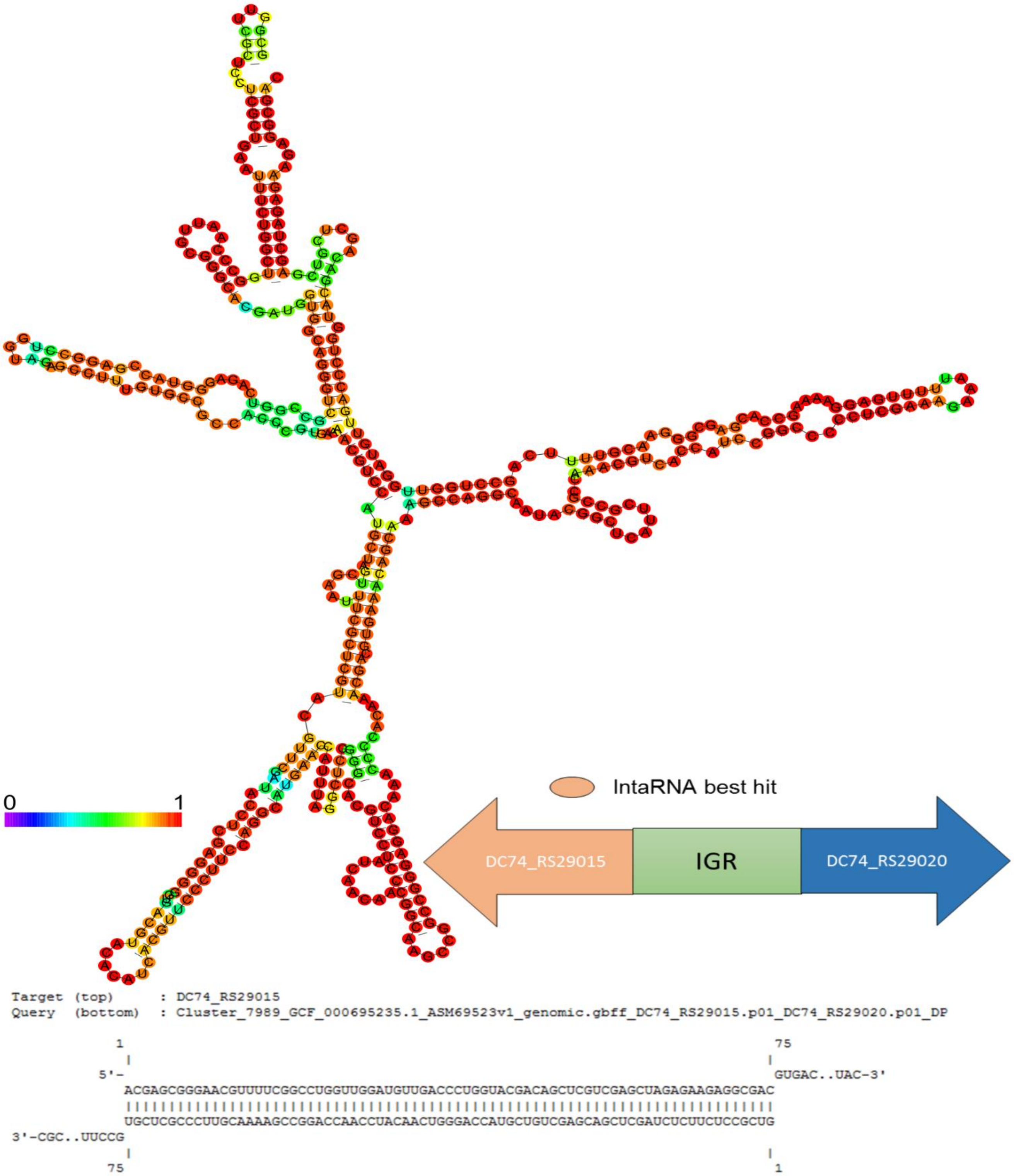


```
Target (top)      : DC74_RS29015
Query  (bottom)   : Cluster_7989_GCF_000695235.1_ASM69523v1_genomic.gbff_DC74_RS29015.p01_DC74_RS29020.p01_DP

            1                                                                          75
            |                                                                          |
          5'-                                                                          GUGAC..UAC-3'
            ACGAGCGGGAACGUUUUCGGCCUGGUUGGAUGUUGACCCUGGUACGACAGCUCGUCGAGCUAGAGAAGAGGCGAC
            |||||||||||||||||||||||||||||||||||||||||||||||||||||||||||||||||||||||||||
            UGCUCGCCCUUGCAAAAGCCGGACCAACCUACAACUGGGACCAUGCUGUCGAGCAGCUCGAUCUCUUCUCCGCUG
3'-CGC..UUCCG
            |                                                                          |
           75                                                                          1
```

Supplementary Figure S7. Secondary structure of a putative non-coding RNA detected by RNAz and located in an intergenic region (IGR) highly conserved in the clade 1 of the *Streptomyces* core genome phylogenetic tree. The consensus minimum free energy (MFE) structure was depicted with RNAfold. The color bar indicates the base-pair probabilities. The flanking genes of the IGR are indicated by arrows. If one of the flanking genes potentially interacts with part of the sequence of the IGR, the arrow is depicted in orange. The region of interaction with the best hit predicted by IntaRNA 2.0 is showed in the bottom of the figure. The representative strain used to detect the possible targets of this IGR was *Streptomyces albulus* NK660.

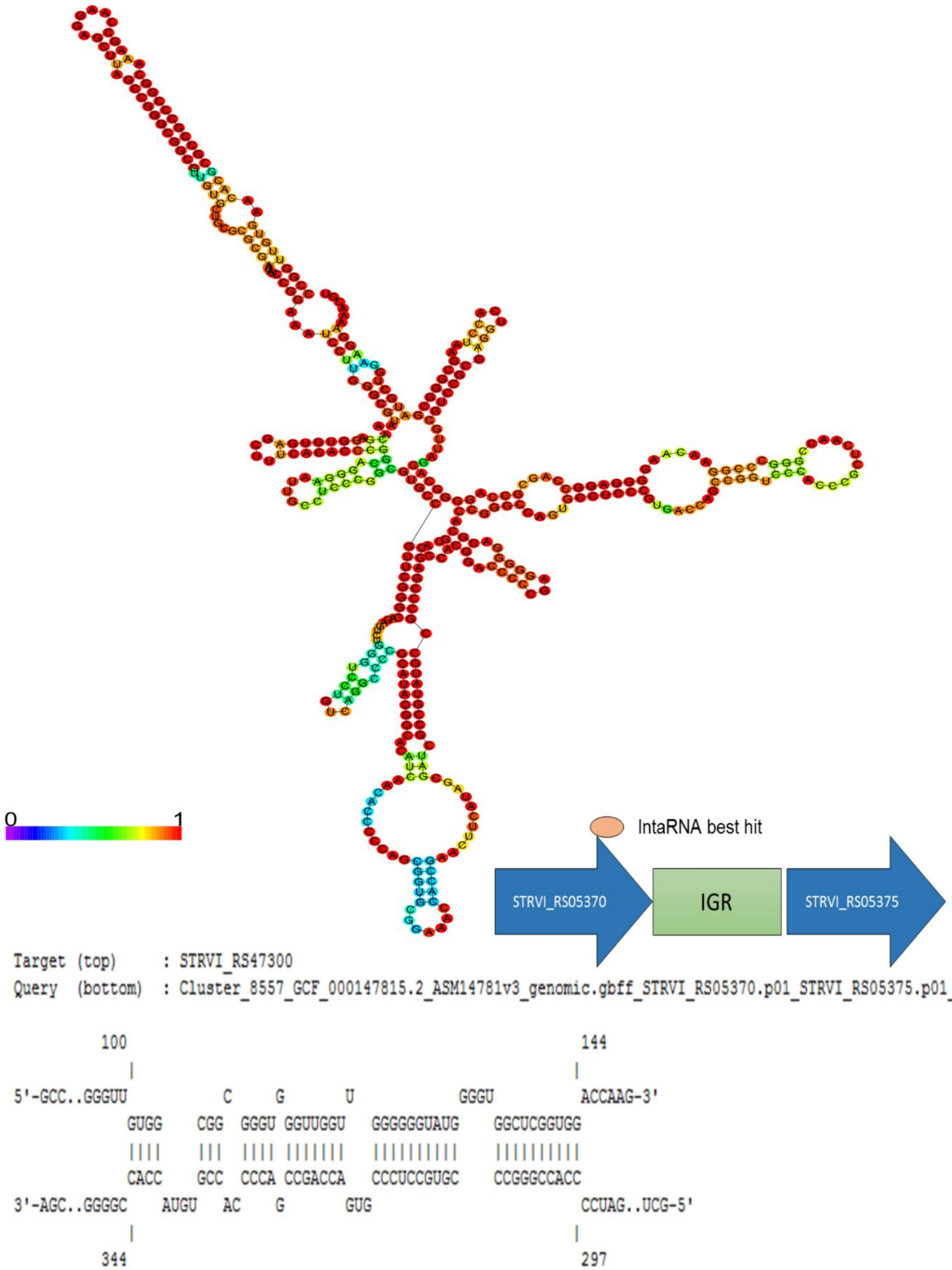


```
Target (top)     : STRVI_RS47300
Query  (bottom)  : Cluster_8557_GCF_000147815.2_ASM14781v3_genomic.gbff_STRVI_RS05370.p01_STRVI_RS05375.p01_CO_F

         100                                           144
           |                                           |
5'-GCC..GGGUU          C     G       U          GGGU          ACCAAG-3'
           GUGG    CGG  GGGU GGUUGGU   GGGGGGUAUG   GGCUCGGUGG
           ||||    |||  |||| |||||||   ||||||||||   ||||||||||
           CACC    GCC  CCCA CCGACCA   CCCUCCGUGC   CCGGGCCACC
3'-AGC..GGGGC   AUGU  AC    G       GUG                        CCUAG..UCG-5'
           |                                           |
         344                                           297
```

Supplementary Figure S8. Secondary structure of a putative non-coding RNA detected by RNAz and located in an intergenic region (IGR) highly conserved in the clade 1 of the *Streptomyces* core genome phylogenetic tree. The consensus minimum free energy (MFE) structure was depicted with RNAfold. The color bar indicates the base-pair probabilities. The flanking genes of the IGR are indicated by arrows. If one of the flanking genes potentially interacts with part of the sequence of the IGR, the arrow is depicted in orange. The region of interaction with the best hit predicted by IntaRNA 2.0 is showed in the bottom of the figure. The representative strain used to detect the possible targets of this IGR was *Streptomyces violaceusniger* Tu 4113.

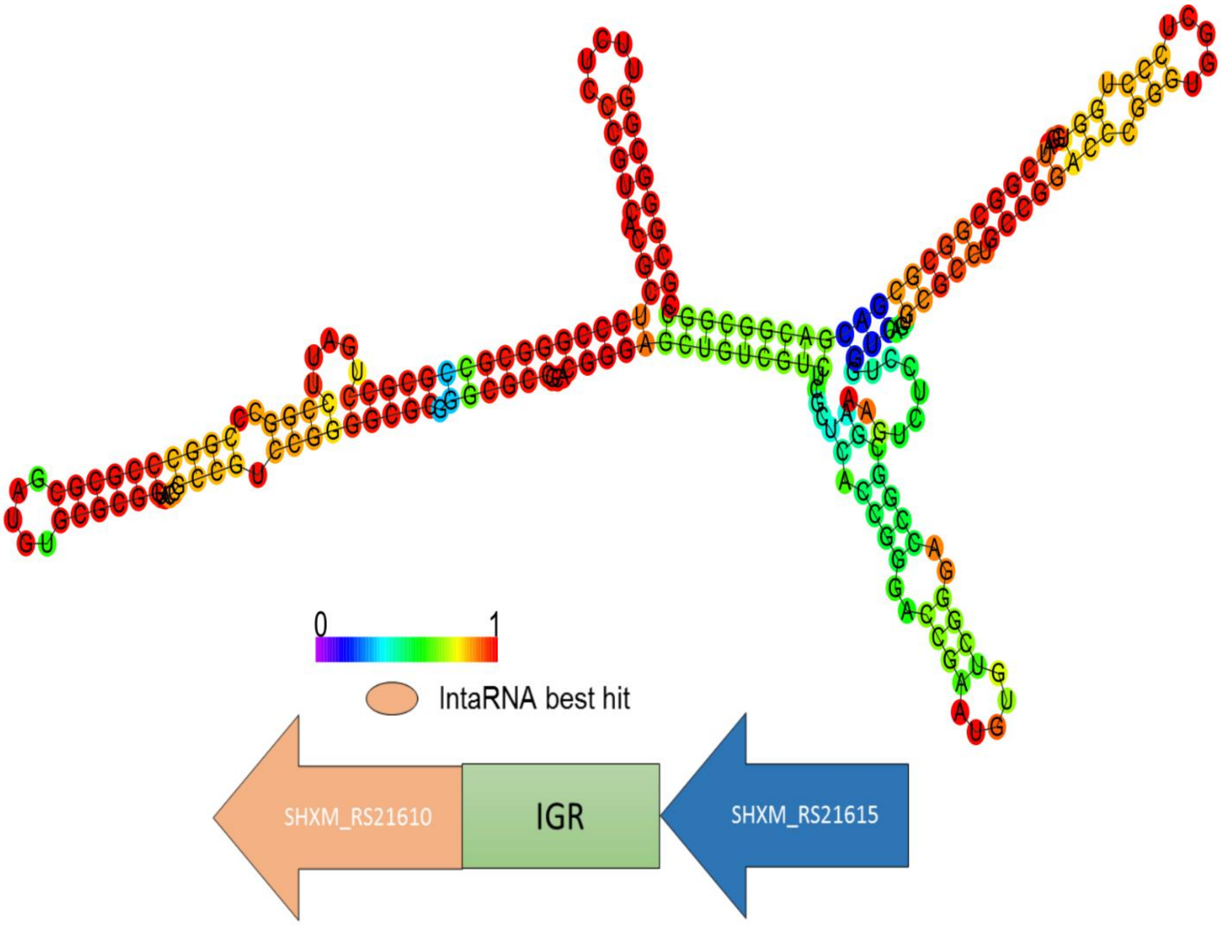


```
Target (top)    : SHXM_RS21610
Query  (bottom) : Cluster_15297_GCF_002021875.1_ASM202187v1_genomic.gbff__SHXM_RS21610.p01_SHXM_RS21615.p01_CO_R

         1                                                                                                                                                      150
         |                                                                                                                                                      |
       5'-
           GACACGCGCACAGUGCGCGGUGGCCGGCCGGAGGCAGGCGCGGCGCCCGGGAGCGUGACGGGAGAAACGCCCGCAUGCUCGAACCAAUUGAGCCGACCCAACCUGUCCAGCCCCUUGAGCCGUCCGCGCCGCGUGCCGCGGCGCCCGGG
           ||||||||||||||||||||||||||||||||||||||||||||||||||||||||||||||||||||||||||||||||||||||||||||||||||||||||||||||||||||||||||||||||||||||||||||||||||||||||
           CUGUGCGCGUGUCACGCGCCCACCGGCCGGCCUCCGUCCGCGCCGCGGGCCCUCGCACUGCCCUCUUUGCGGGCGUACGAGCUUGGUUAACUCGGCUGGGUUGGACAGGUCGGGGAACUCGGCAGGCGCGGCGCACGGCGCCGCGGGCCC
3'-CCG..CCGCC                                                                                                                                                       CUAGGG-5'
         |                                                                                                                                                      |
       156                                                                                                                                                      7
```

Supplementary Figure S9. Secondary structure of a putative non-coding RNA detected by RNAz and located in an intergenic region (IGR) highly conserved in the clade 1 of the *Streptomyces* core genome phylogenetic tree. The consensus minimum free energy (MFE) structure was depicted with RNAfold. The color bar indicates the base-pair probabilities. The flanking genes of the IGR are indicated by arrows. If one of the flanking genes potentially interacts with part of the sequence of the IGR, the arrow is depicted in orange. The region of interaction with the best hit predicted by IntaRNA 2.0 is showed in the bottom of the figure. The representative strain used to detect the possible targets of this IGR was *Streptomyces hygroscopicus* XM201.

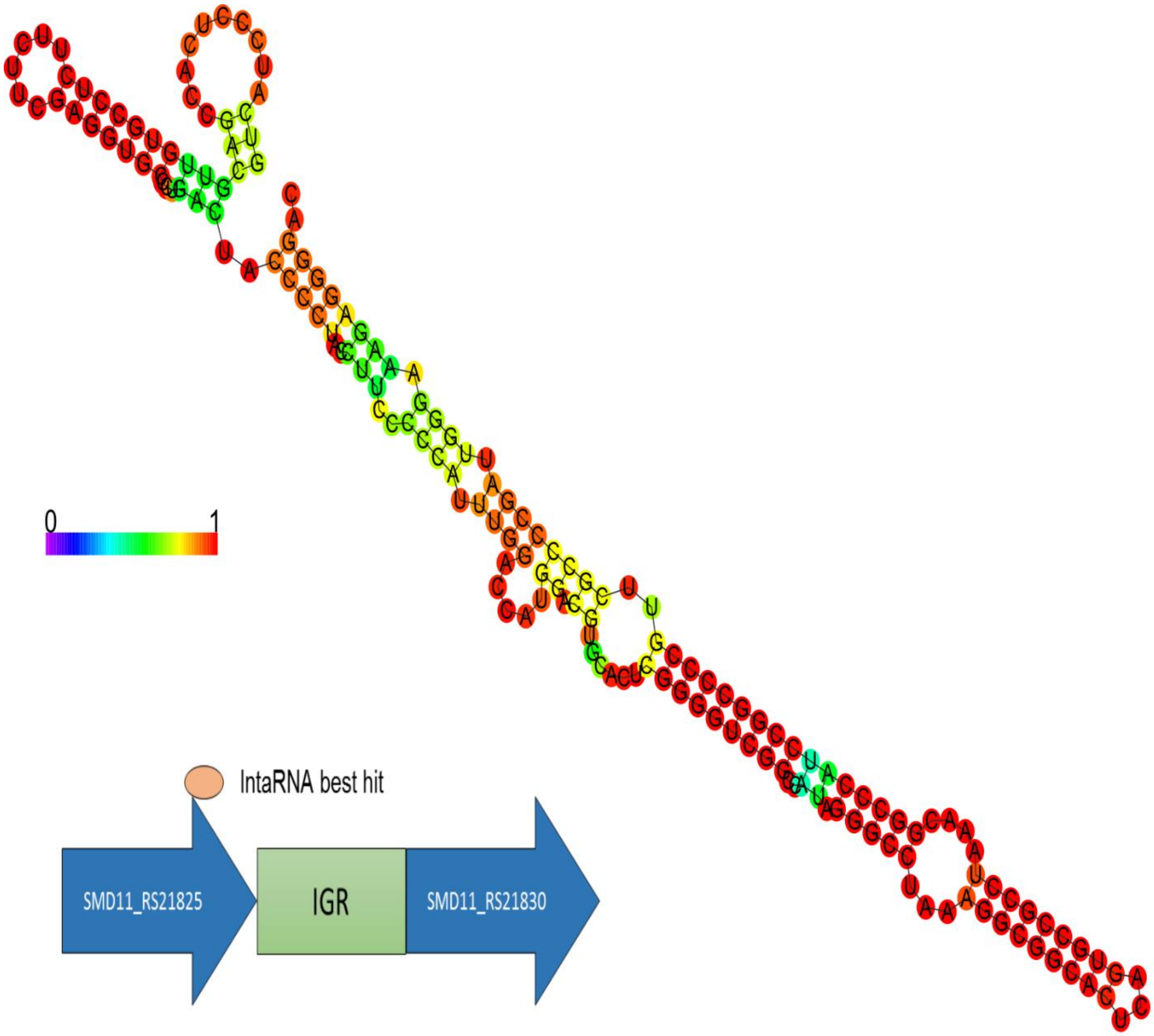


```
Target (top)     : SMD11_RS15435
Query  (bottom)  : Cluster_25022_GCF_002192455.1_ASM219245v1_genomic.gbff_SMD11_RS21825.p01_SMD11_RS21830.p01_CO_F

          84                                                                150
           |                                                                 |
5'-CUG..ACGAC           GCUACC     C      G    CC    G   CC    CAGGUG    GA
             CCC CGCCGACG      GUGCC UCUUCU CGA  UGC CU  GAC      ACC  UGCCUUCUCC
             ||| ||||||||      ||||| |||||| |||  ||| ||  |||      |||  ||||||||||
             GGG GUGGCUGC      CACGG AGAAGA GCU  ACG GA  CUG      UGG  AUGGAAGGGG
     3'-CAGUA   A       AA               A    CC   G       A        GG          GUAAA..AGG-5'
           |                                                                 |
          208                                                               153
```

Supplementary Figure S10. Secondary structure of a putative non-coding RNA detected by RNAz and located in an intergenic region (IGR) highly conserved in the clade 1 of the *Streptomyces* core genome phylogenetic tree. The consensus minimum free energy (MFE) structure was depicted with RNAfold. The color bar indicates the base-pair probabilities. The flanking genes of the IGR are indicated by arrows. If one of the flanking genes potentially interacts with part of the sequence of the IGR, the arrow is depicted in orange. The region of interaction with the best hit predicted by IntaRNA 2.0 is showed in the bottom of the figure. The representative strain used to detect the possible targets of this IGR was *Streptomyces albireticuli* MDJK11.

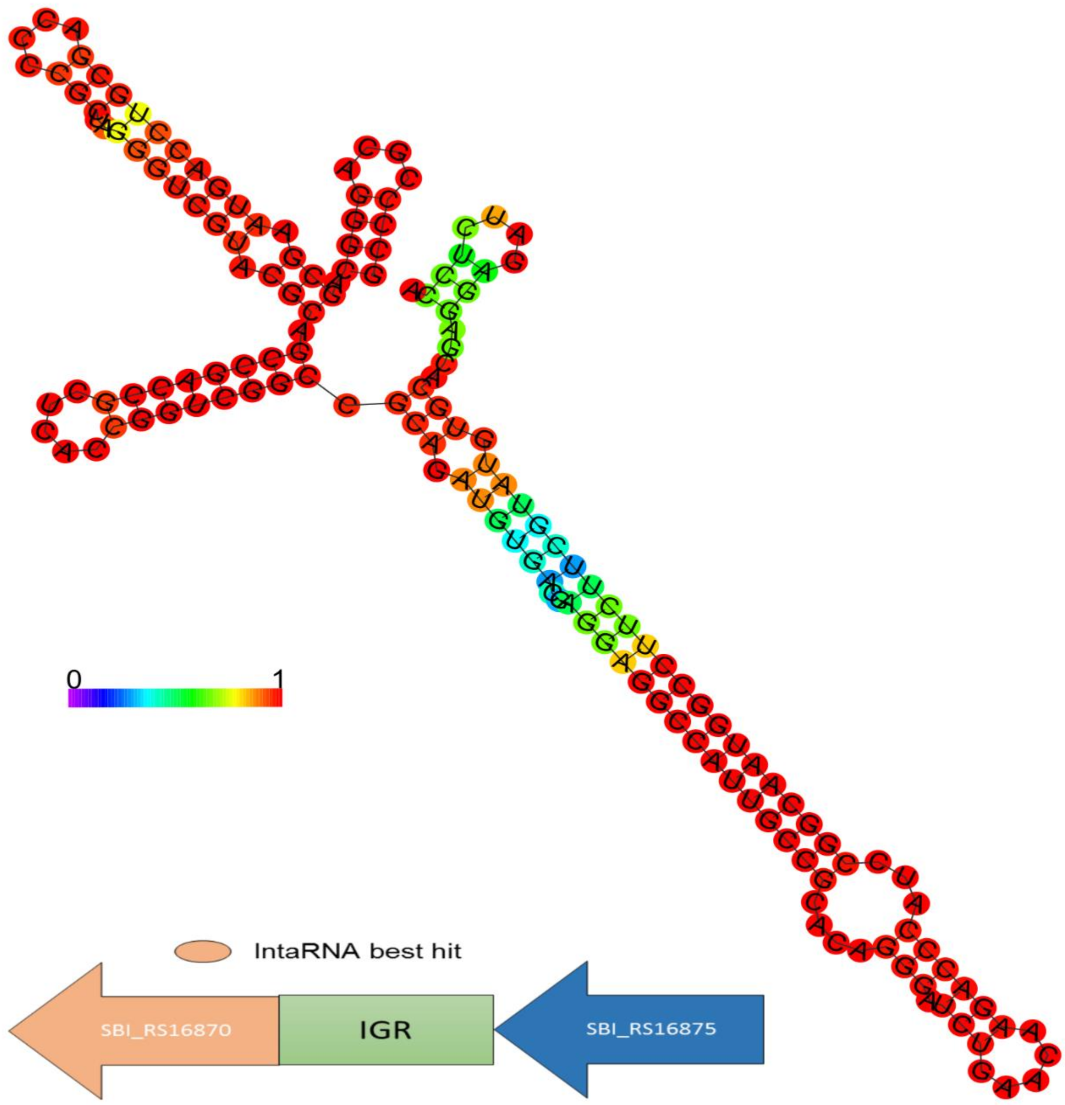


```
Target (top)     : SBI_RS16870
Query  (bottom)  : Cluster_29600_GCF_000092385.1_ASM9238v1_genomic.gbff_SBI_RS16870.p01_SBI_RS16875.p01_CO_R

            1                                                                          75
            |                                                                          |
         5'-                                                                           AUGAA..UUC-3'
            AGGCCAUUGCCGCAGAGGUCGAACAAGGACCCGUCCGGCAAUGGCCUUCUUCGUAUGUGCACGAGGAGAUCUCCA
            |||||||||||||||||||||||||||||||||||||||||||||||||||||||||||||||||||||||||||
            UCCGGUAACGGCGUCUCCAGCUUGUUCCUGGGCAGGCCGUUACCGGAAGAAGCAUACACGUGCUCCUCUAGAGGU
 3'-CGC..GCUCC
            |                                                                          |
           75                                                                          1
```

Supplementary Figure S11. Secondary structure of a putative non-coding RNA detected by RNAz and located in an intergenic region (IGR) highly conserved in the clade 1 of the *Streptomyces* core genome phylogenetic tree. The consensus minimum free energy (MFE) structure was depicted with RNAfold. The color bar indicates the base-pair probabilities. The flanking genes of the IGR are indicated by arrows. If one of the flanking genes potentially interacts with part of the sequence of the IGR, the arrow is depicted in orange. The region of interaction with the best hit predicted by IntaRNA 2.0 is showed in the bottom of the figure. The representative strain used to detect the possible targets of this IGR was *Streptomyces bingchenggensis* BCW-1.

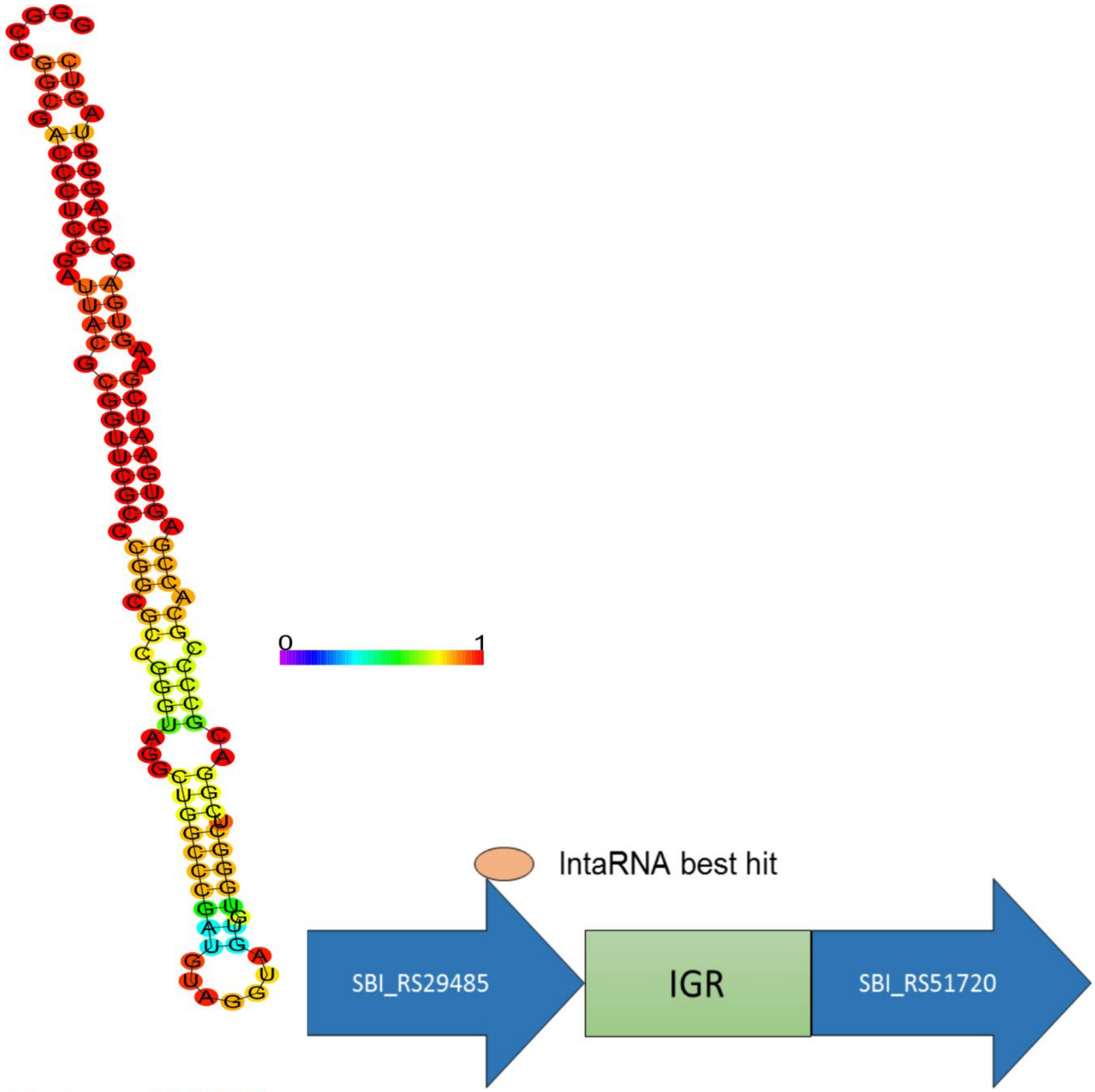


```
Target (top)     : SBI_RS35760
Query  (bottom)  : Cluster_38357_GCF_000092385.1_ASM9238v1_genomic.gbff_SBI_RS29485.p01_SBI_RS51720.p01_CO_F

          117                              144
            |                              |
5'-GAC..GCCAC            CAUCC             AUCGGC-3'
             CGCCCCG CCCCU     UC GGCCCGUCC
             ||||||| |||||     || |||||||||
             GCGGGGC GGGGA     AG CCGGGCAGG
3'-CUG..UCCCC       C     CGAC   U         CCCG-5'
            |                              |
           33                              5
```

Supplementary Figure S12. Secondary structure of a putative non-coding RNA detected by RNAz and located in an intergenic region (IGR) highly conserved in the clade 1 of the *Streptomyces* core genome phylogenetic tree. The consensus minimum free energy (MFE) structure was depicted with RNAfold. The color bar indicates the base-pair probabilities. The flanking genes of the IGR are indicated by arrows. If one of the flanking genes potentially interacts with part of the sequence of the IGR, the arrow is depicted in orange. The region of interaction with the best hit predicted by IntaRNA 2.0 is showed in the bottom of the figure. The representative strain used to detect the possible targets of this IGR was *Streptomyces bingchenggensis* BCW-1.

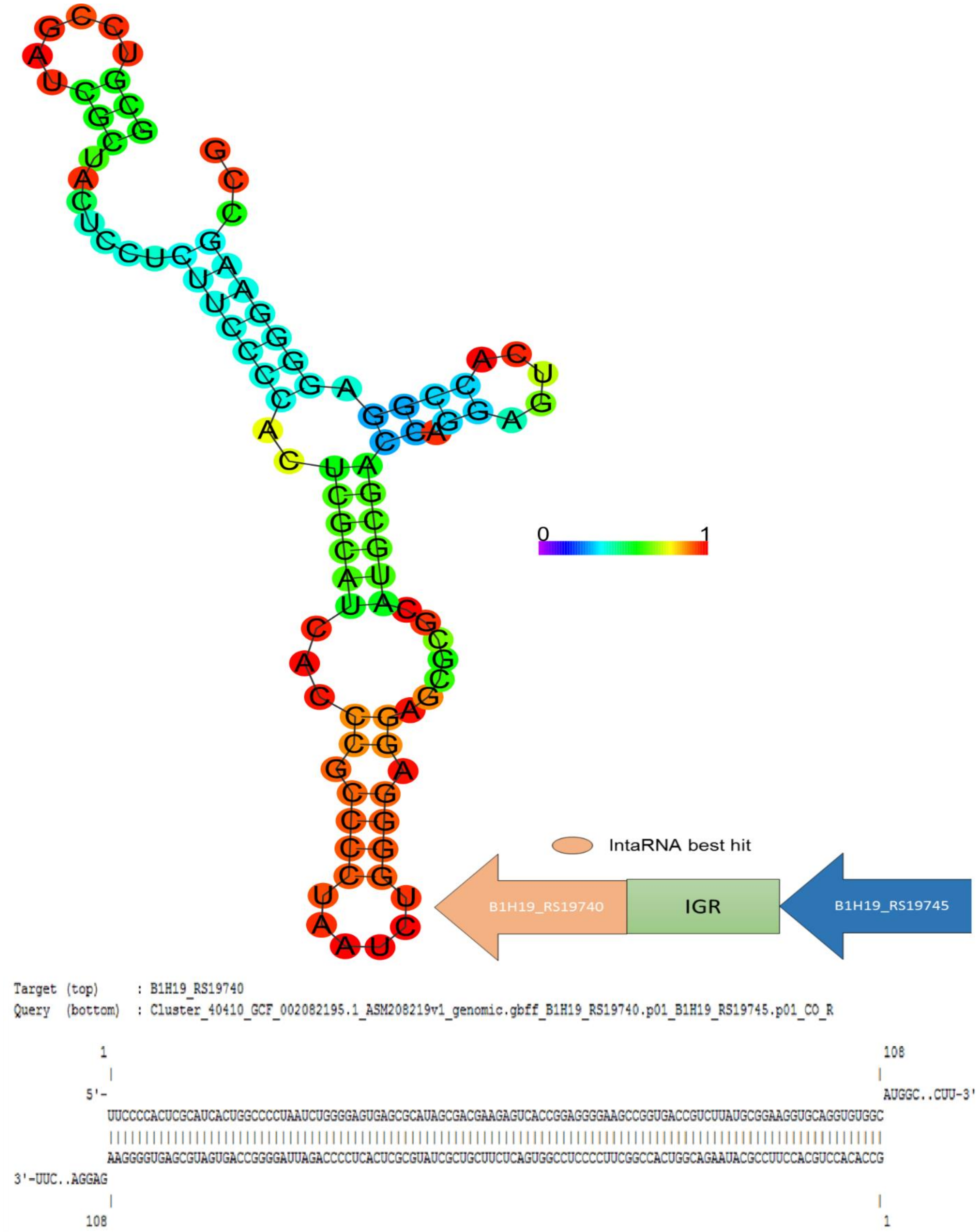

Supplementary Figure S13. Secondary structure of a putative non-coding RNA detected by RNAz and located in an intergenic region (IGR) highly conserved in the clade 1 of the *Streptomyces* core genome phylogenetic tree. The consensus minimum free energy (MFE) structure was depicted with RNAfold. The color bar indicates the base-pair probabilities. The flanking genes of the IGR are indicated by arrows. If one of the flanking genes potentially interacts with part of the sequence of the IGR, the arrow is depicted in orange. The region of interaction with the best hit predicted by IntaRNA 2.0 is showed in the bottom of the figure. The representative strain used to detect the possible targets of this IGR was *Streptomyces gilvosporeus* F607.

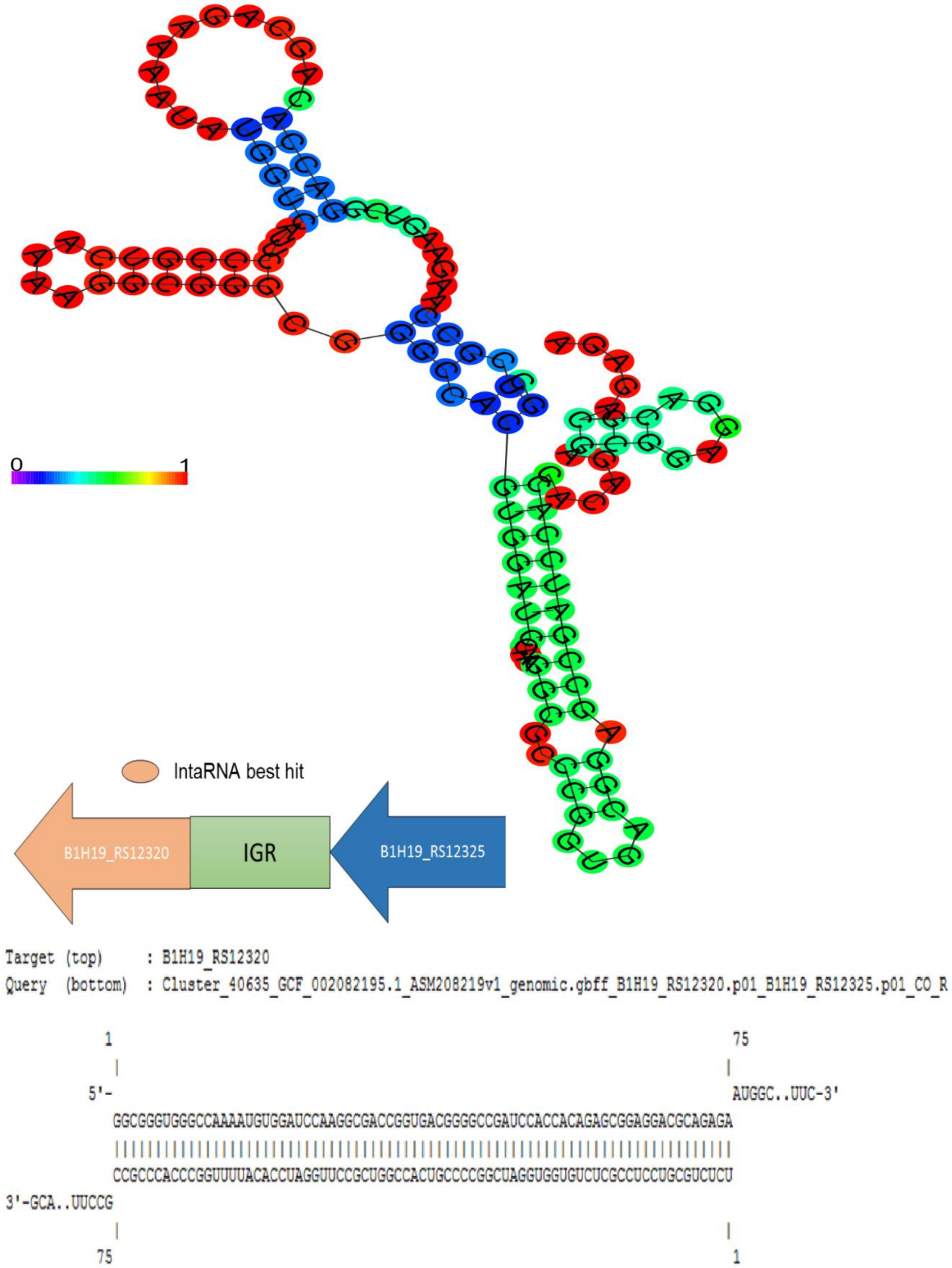

Supplementary Figure S14. Secondary structure of a putative non-coding RNA detected by RNAz and located in an intergenic region (IGR) highly conserved in the clade 1 of the *Streptomyces* core genome phylogenetic tree. The consensus minimum free energy (MFE) structure was depicted with RNAfold. The color bar indicates the base-pair probabilities. The flanking genes of the IGR are indicated by arrows. If one of the flanking genes potentially interacts with part of the sequence of the IGR, the arrow is depicted in orange. The region of interaction with the best hit predicted by IntaRNA 2.0 is showed in the bottom of the figure. The representative strain used to detect the possible targets of this IGR was *Streptomyces gilvosporeus* F607.

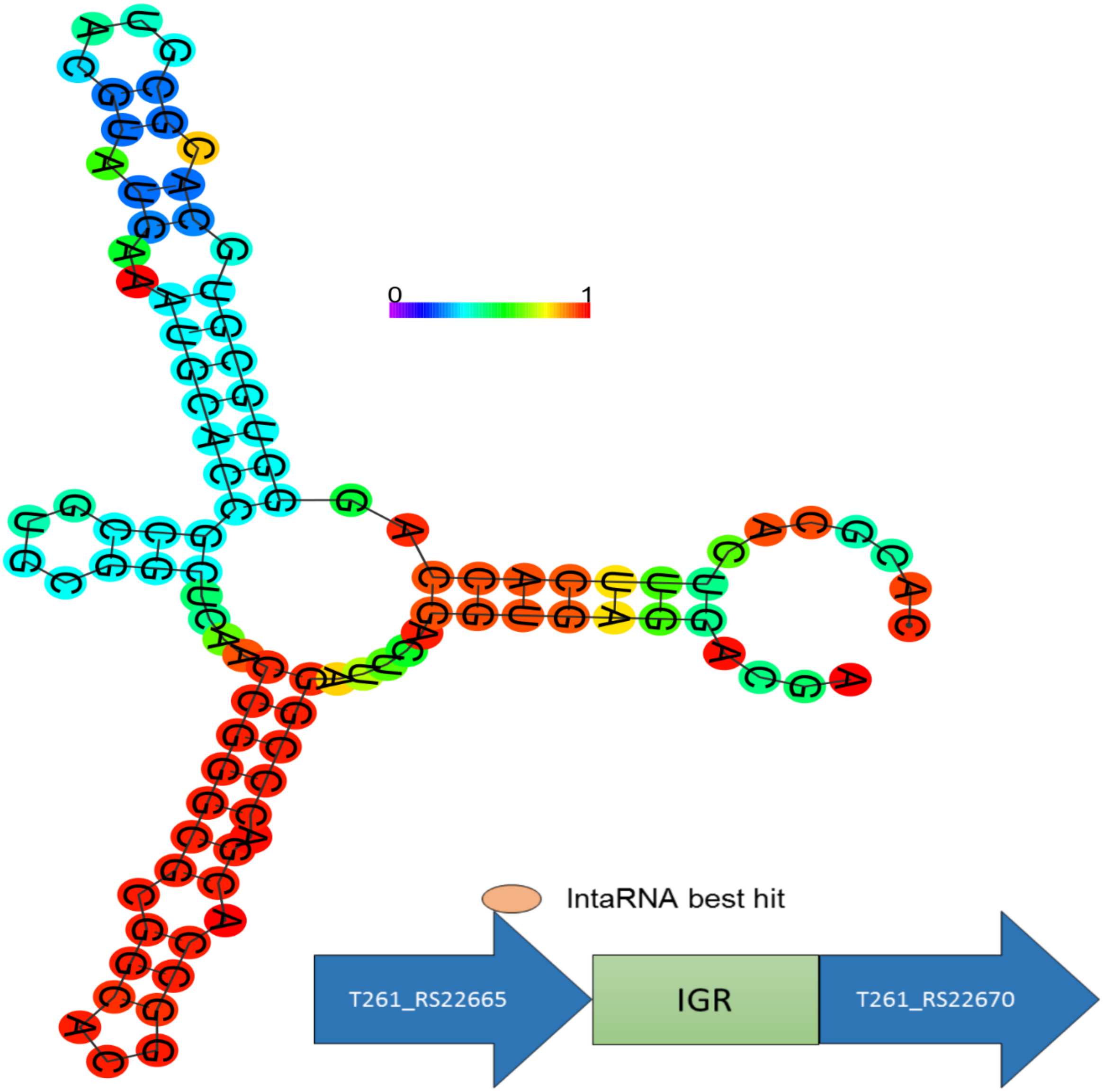


```
Target (top)     : T261_RS04625
Query  (bottom)  : Cluster_46825_GCF_000952035.2_ASM95203v2_genomic.gbff_T261_RS22665.r01_T261_RS22670.p01_CO_F

        111                              145
          |                              |
5'-UGU..CUUCC       CCCU   GCAAA  CACA          ACUAC-3'
             CGUCCUC    ACC     CC     CGUGGCCGUG
             |||||||    |||     ||     ||||||||||
             GCAGGAG    UGG     GG     GCACCGGCAC
        3'-A               ACUUA  CCCA          GGCGC..CAC-5'
          |                              |
        105                              75
```

Supplementary Figure S15. Secondary structure of a putative non-coding RNA detected by RNAz and located in an intergenic region (IGR) highly conserved in the clade 1 of the *Streptomyces* core genome phylogenetic tree. The consensus minimum free energy (MFE) structure was depicted with RNAfold. The color bar indicates the base-pair probabilities. The flanking genes of the IGR are indicated by arrows. If one of the flanking genes potentially interacts with part of the sequence of the IGR, the arrow is depicted in orange. The region of interaction with the best hit predicted by IntaRNA 2.0 is showed in the bottom of the figure. The representative strain used to detect the possible targets of this IGR was *Streptomyces lydicus* A02.

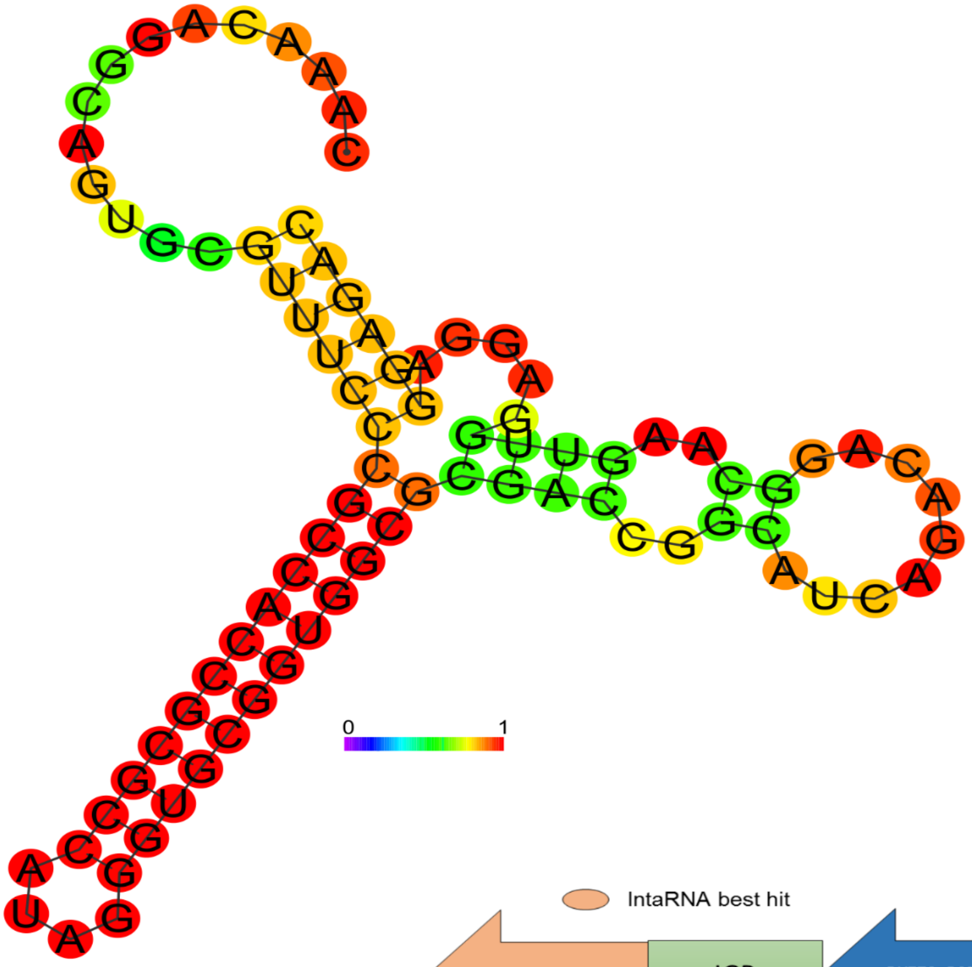

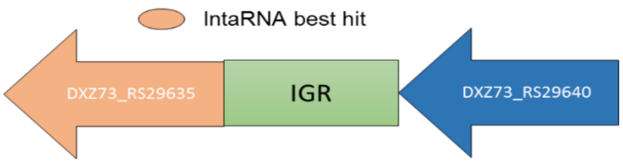


```
Target (top)     : DXZ73_RS29635
Query  (bottom)  : Cluster_52847_GCF_003391135.1_ASM339113v1_genomic.gbff_DXZ73_RS29635.p01_DXZ73_RS29640.p01_CO_R

          1                                                                          75
           |                                                                          |
        5'-                                                                          GUGGC..UUC-3'
           UGCGUUUCGCGCCACCGCGCCAUAAGGUGCGGUGGCGCGACCAGCAGCAGACAGGCGAGUUGGAGGAGGAUCGAC
           |||||||||||||||||||||||||||||||||||||||||||||||||||||||||||||||||||||||||||
           ACGCAAAGCGCGGUGGCGCGGUAUUCCACGCCACCGCGCUGGUCGUCGUCUGUCCGCUCAACCUCCUCCUAGCUG
3'-GUU..CCGUC
           |                                                                          |
         75                                                                           1
```

Supplementary Figure S16. Secondary structure of a putative non-coding RNA detected by RNAz and located in an intergenic region (IGR) highly conserved in the clade 1 of the *Streptomyces* core genome phylogenetic tree. The consensus minimum free energy (MFE) structure was depicted with RNAfold. The color bar indicates the base-pair probabilities. The flanking genes of the IGR are indicated by arrows. If one of the flanking genes potentially interacts with part of the sequence of the IGR, the arrow is depicted in orange. The region of interaction with the best hit predicted by IntaRNA 2.0 is showed in the bottom of the figure. The representative strain used to detect the possible targets of this IGR was *Streptomyces olivoreticuli olivoreticuli* ATCC 31159.

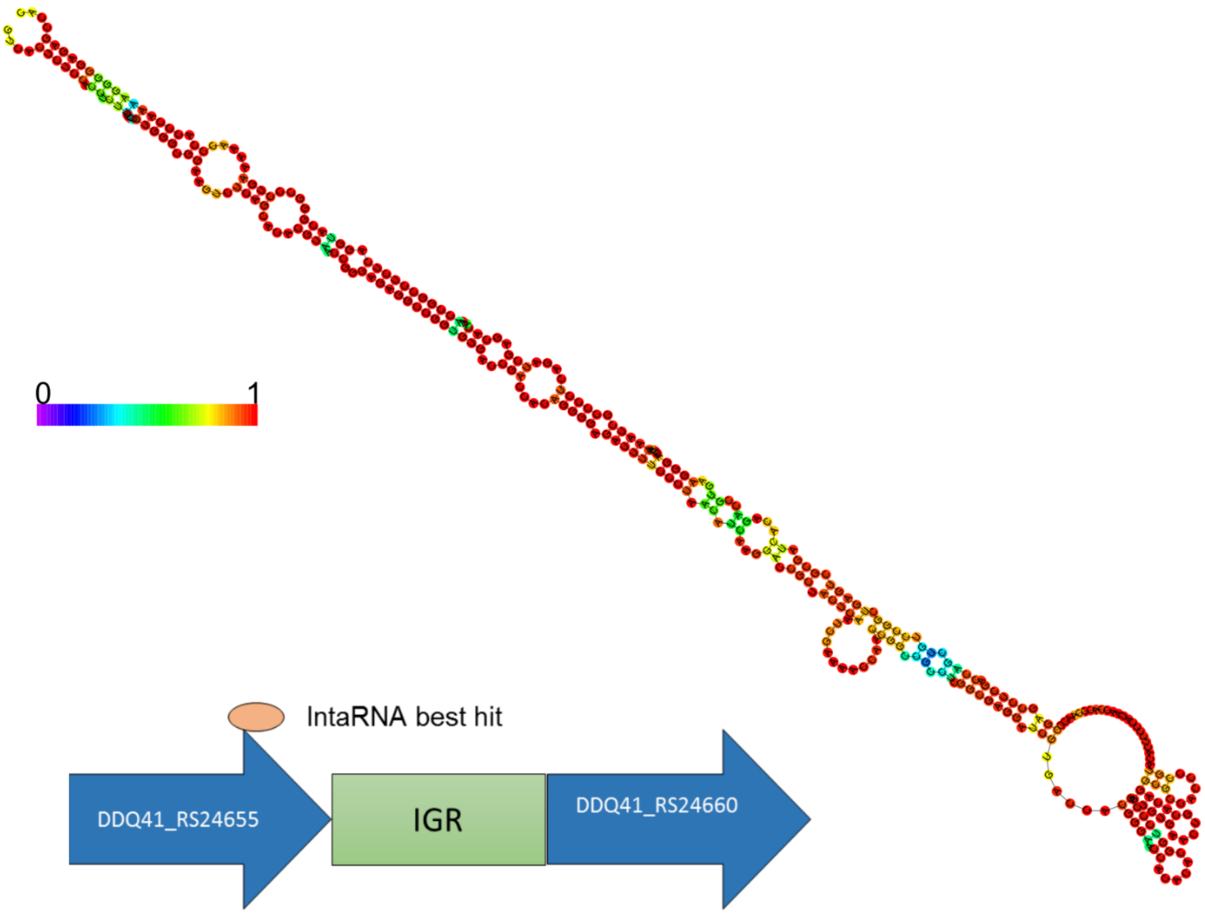


```
Target (top)    : DDQ41_RS24645
Query  (bottom) : Cluster_12890_GCF_003122365.1_ASM312236v1_genomic.gbff_DDQ41_RS24655.p01_DDQ41_RS24660.p01_CO_F

          57                                                     112
           |                                                      |
5'-UGA..AGCCA     C     A          ACCGAUCCC   CCGC   C           UCCCC..GCG-3'
             CGCAC CGGG GCCACUCCGUG         UGC    CCCG UC  UCCGACCCCC
             |||||  |||| |||||||||||         |||    |||| ||  ||||||||||
             GCGUG GCCC CGGUGGGCAC          AUG    GGGC AG  AGGCUGGGGG
3'-CAG..UUACA     AAGU  A                       AA         UA         CCUUU..CGC-5'
           |                                                      |
          234                                                    187
```

Supplementary Figure S17. Secondary structure of a putative non-coding RNA detected by RNAz and located in an intergenic region (IGR) highly conserved in the clade 2 of the *Streptomyces* core genome phylogenetic tree. The consensus minimum free energy (MFE) structure was depicted with RNAfold. The color bar indicates the base-pair probabilities. The flanking genes of the IGR are indicated by arrows. If one of the flanking genes potentially interacts with part of the sequence of the IGR, the arrow is depicted in orange. The region of interaction with the best hit predicted by IntaRNA 2.0 is showed in the bottom of the figure. The representative strain used to detect the possible targets of this IGR was *Streptomyces spongiicola* HNM0071.

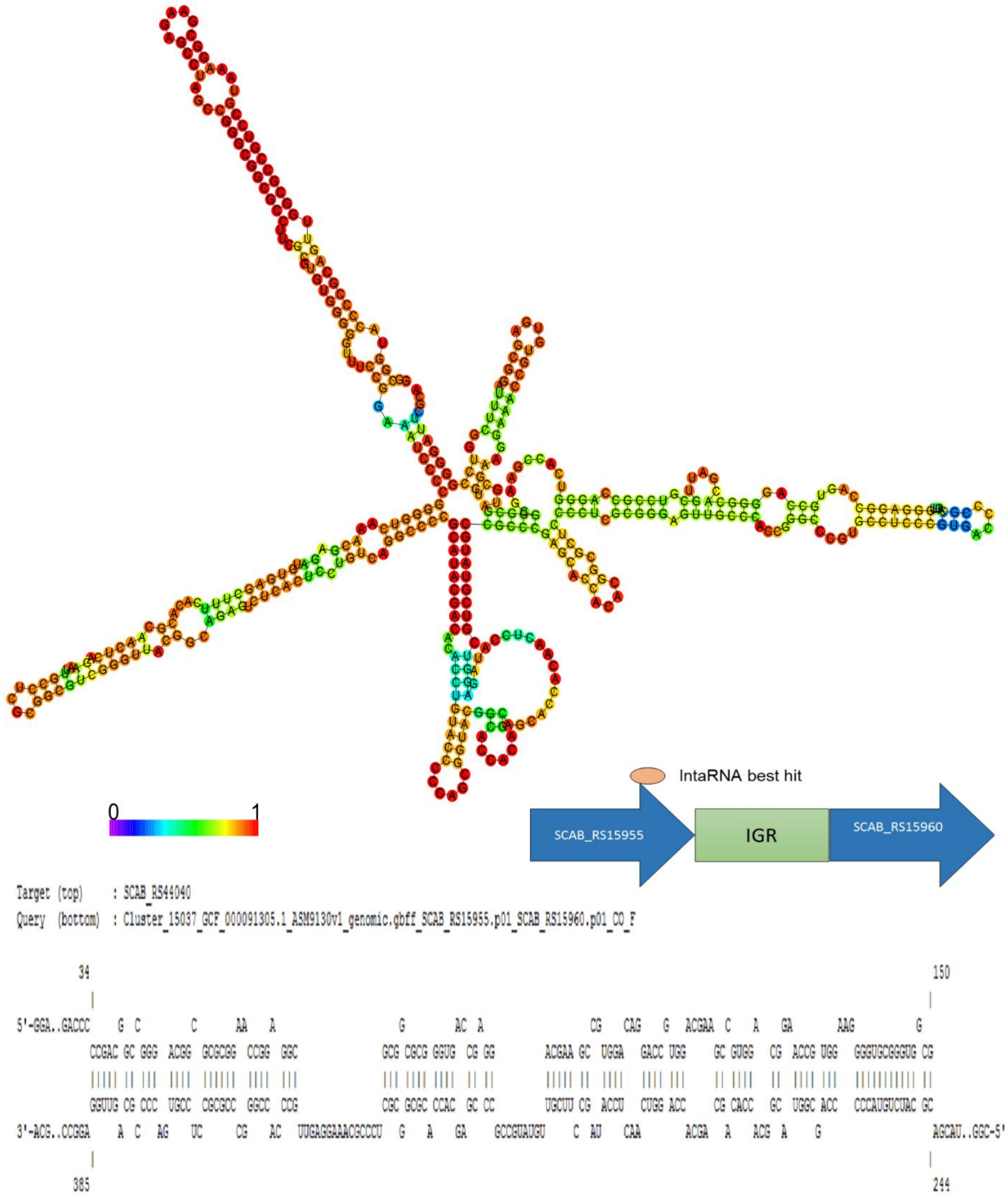

Supplementary Figure S18. Secondary structure of a putative non-coding RNA detected by RNAz and located in an intergenic region (IGR) highly conserved in the clade 3 of the *Streptomyces* core genome phylogenetic tree. The consensus minimum free energy (MFE) structure was depicted with RNAfold. The color bar indicates the base-pair probabilities. The flanking genes of the IGR are indicated by arrows. If one of the flanking genes potentially interacts with part of the sequence of the IGR, the arrow is depicted in orange. The region of interaction with the best hit predicted by IntaRNA 2.0 is showed in the bottom of the figure. The representative strain used to detect the possible targets of this IGR was *Streptomyces scabiei* 87.22.

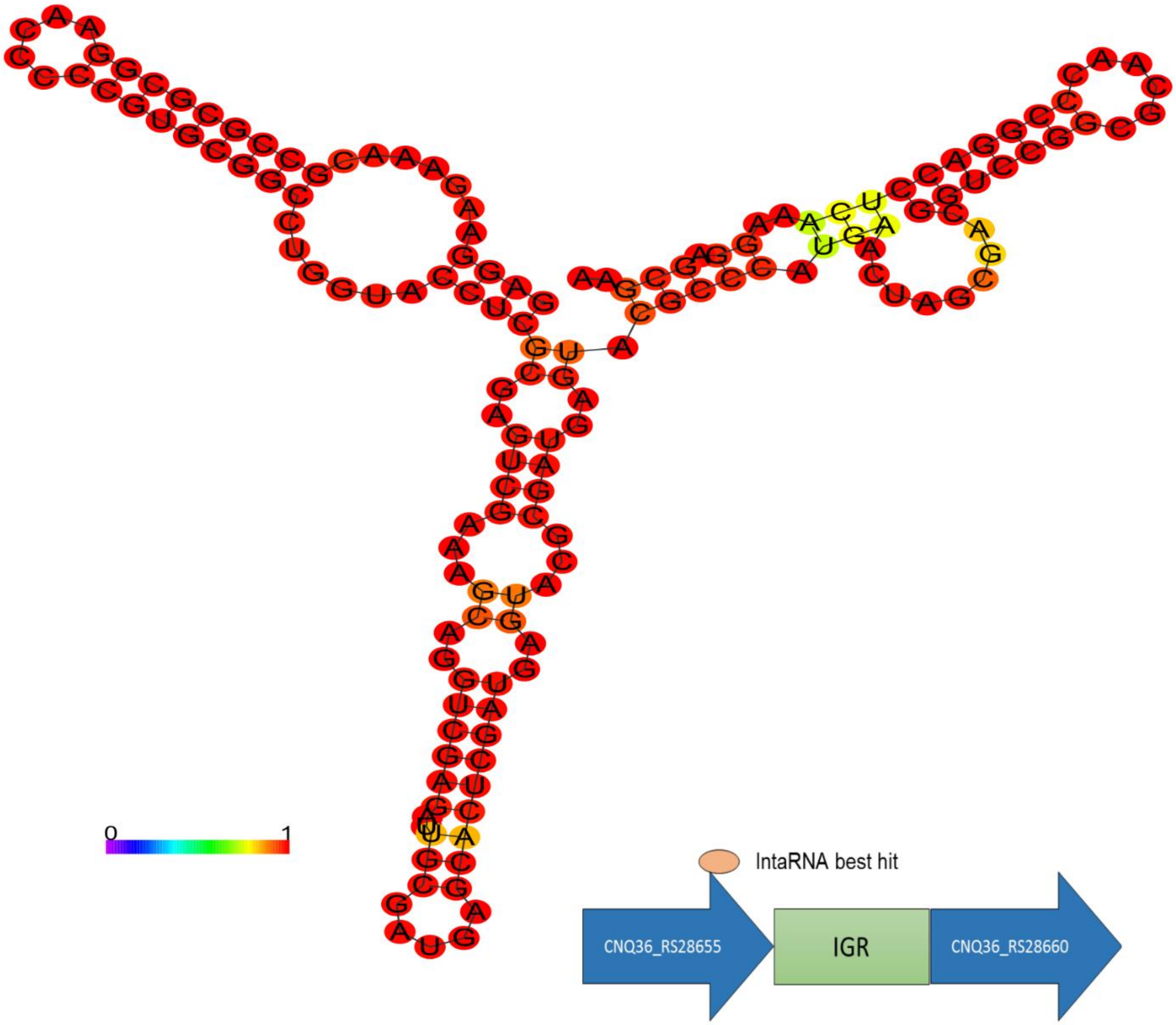


```
Target (top)     : CNQ36_RS14660
Query  (bottom)  : Cluster_73940_GCF_003665435.1_ASM366543v1_genomic.gbff_CNQ36_RS28655.p01_CNQ36_RS28660.p01_CO_F

          11                                          50
           |                                          |
5'-GGACUGAAUC     AA      ACUAU            C          AGUGA..GCC-3'
           CCGGG  UGCGUC     GGACCGUCGUUAG         ACUCAUCG
           |||||  ||||||     |||||||||||||         ||||||||
           GGCCC  ACGCAG     CCUGGCAGCGAUC         UGAGUAGC
3'-AAG..CUCCA     A       G            AAGUACCCGCA          GCAUG..ACG-5'
           |                                          |
          168                                        124
```

Supplementary Figure S19. Secondary structure of a putative non-coding RNA detected by RNAz and located in an intergenic region (IGR) highly conserved in the clade 3 of the *Streptomyces* core genome phylogenetic tree. The consensus minimum free energy (MFE) structure was depicted with RNAfold. The color bar indicates the base-pair probabilities. The flanking genes of the IGR are indicated by arrows. If one of the flanking genes potentially interacts with part of the sequence of the IGR, the arrow is depicted in orange. The region of interaction with the best hit predicted by IntaRNA 2.0 is showed in the bottom of the figure. The representative strain used to detect the possible targets of this IGR was *Streptomyces fungicidicus* TXX3120.

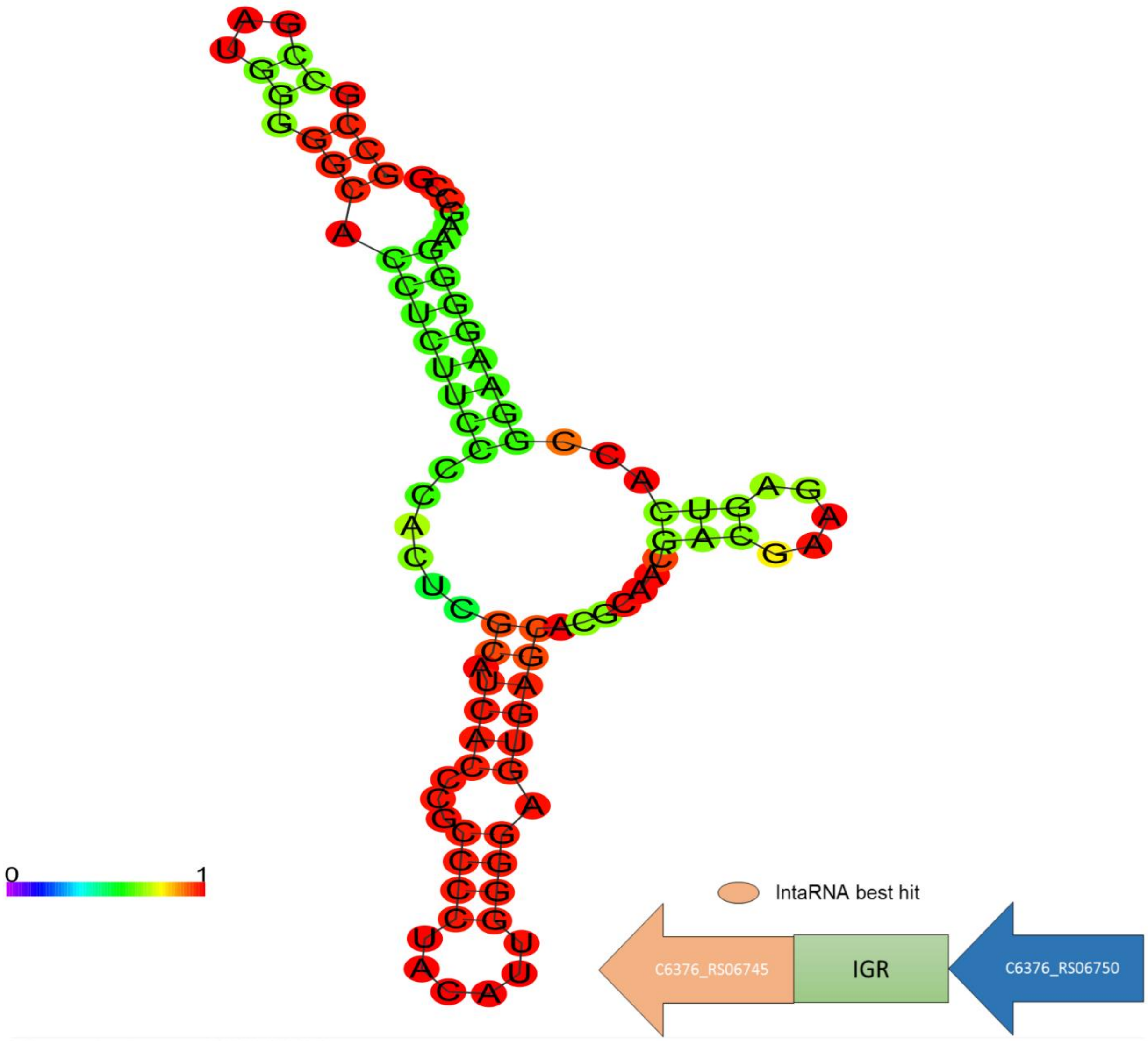


```
Target (top)     : C6376_RS06745
Query  (bottom)  : Cluster_102279_GCF_003032475.1_ASM303247v1_genomic.gbff_C6376_RS06745.p01_C6376_RS06750.p01_CO_R

          1                                                                           75
          |                                                                           |
        5'-                                                                           AUGGC..CGA-3'
           GCACGCAGCGACGAAGAGUCACCGGAAGGGGAAGCCGGUGGCCGUCGAGUCGAGUGCGGAAGGUUCAGGUGUGUC
           |||||||||||||||||||||||||||||||||||||||||||||||||||||||||||||||||||||||||||
           CGUGCGUCGCUGCUUCUCAGUGGCCUUCCCCUUCGGCCACCGGCAGCUCAGCUCACGCCUUCCAAGUCCACACAG
3'-CGG..UCACU
          |                                                                           |
         75                                                                           1
```

Supplementary Figure S20. Secondary structure of a putative non-coding RNA detected by RNAz and located in an intergenic region (IGR) highly conserved in the clade 3 of the *Streptomyces* core genome phylogenetic tree. The consensus minimum free energy (MFE) structure was depicted with RNAfold. The color bar indicates the base-pair probabilities. The flanking genes of the IGR are indicated by arrows. If one of the flanking genes potentially interacts with part of the sequence of the IGR, the arrow is depicted in orange. The region of interaction with the best hit predicted by IntaRNA 2.0 is showed in the bottom of the figure. The representative strain used to detect the possible targets of this IGR was *Streptomyces* sp. P3.

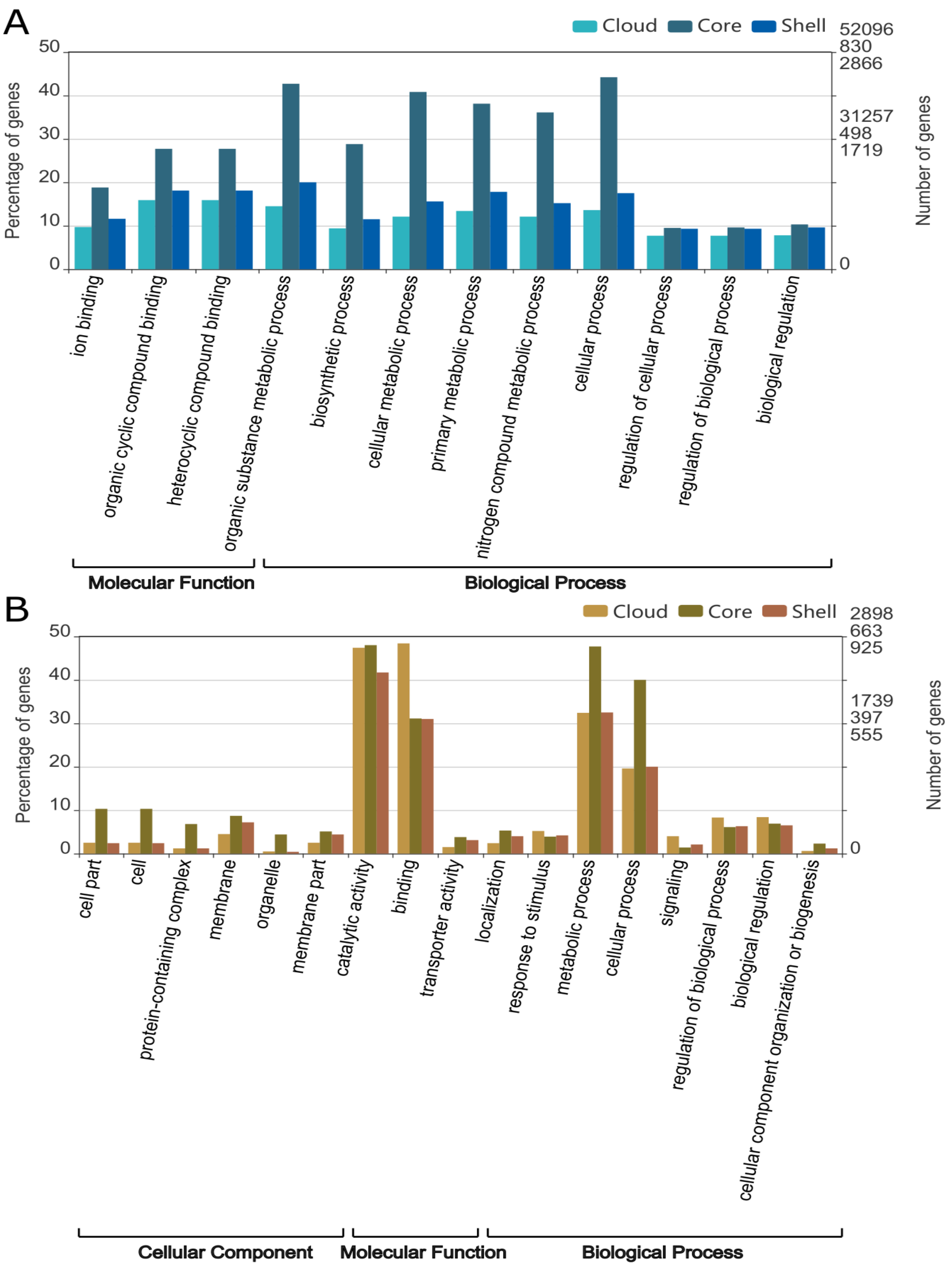

Supplementary Figure S21. Functional description of the categories of the pan-genome for **(A)** Roary and **(B)** Micropan by annotations of Gene Ontology (GO). Only the categories with significant differences (p-value < 0.01) in each pan-genome category after a Pearson's chi-squared test of homogeneity were depicted in the plot. "Core" label includes both core and soft-core genome.

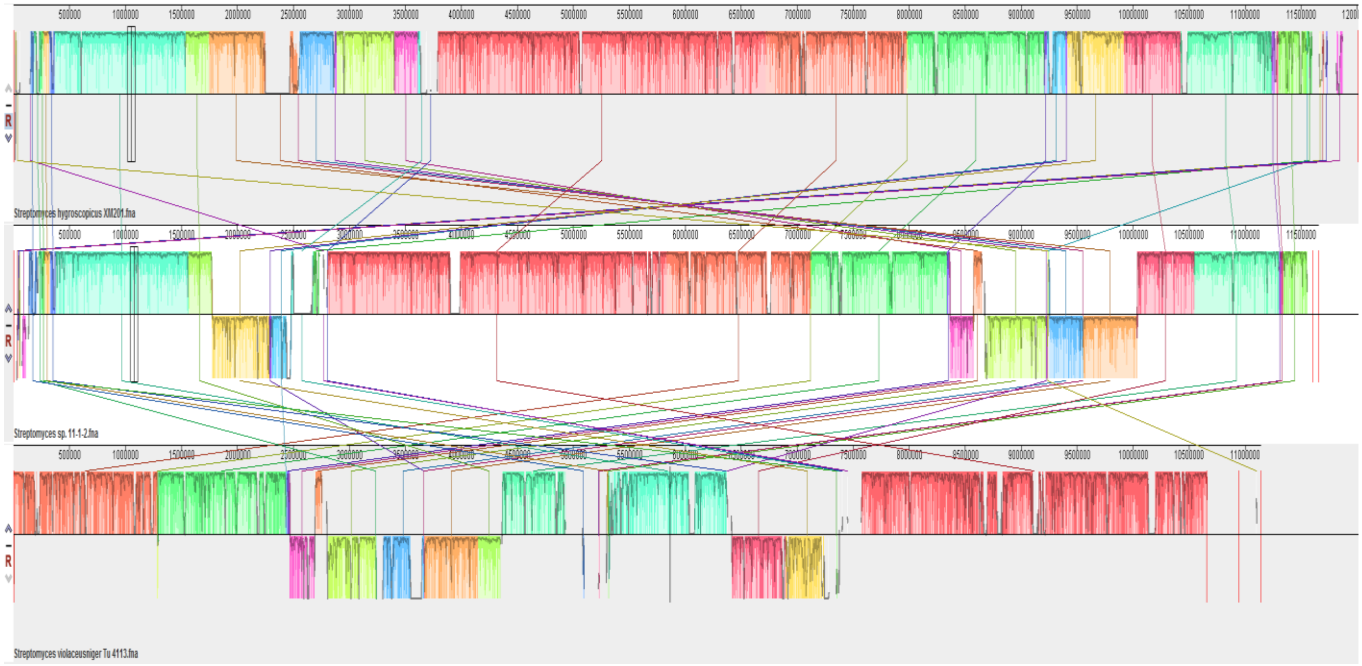

Supplementary Figure S22. Multiple genome alignment performed with the progressiveMauve algorithm for *S. hygroscopicus* XM201, *Streptomyces* sp. 11-1-2 and *S. violaceusniger* Tu 4113.

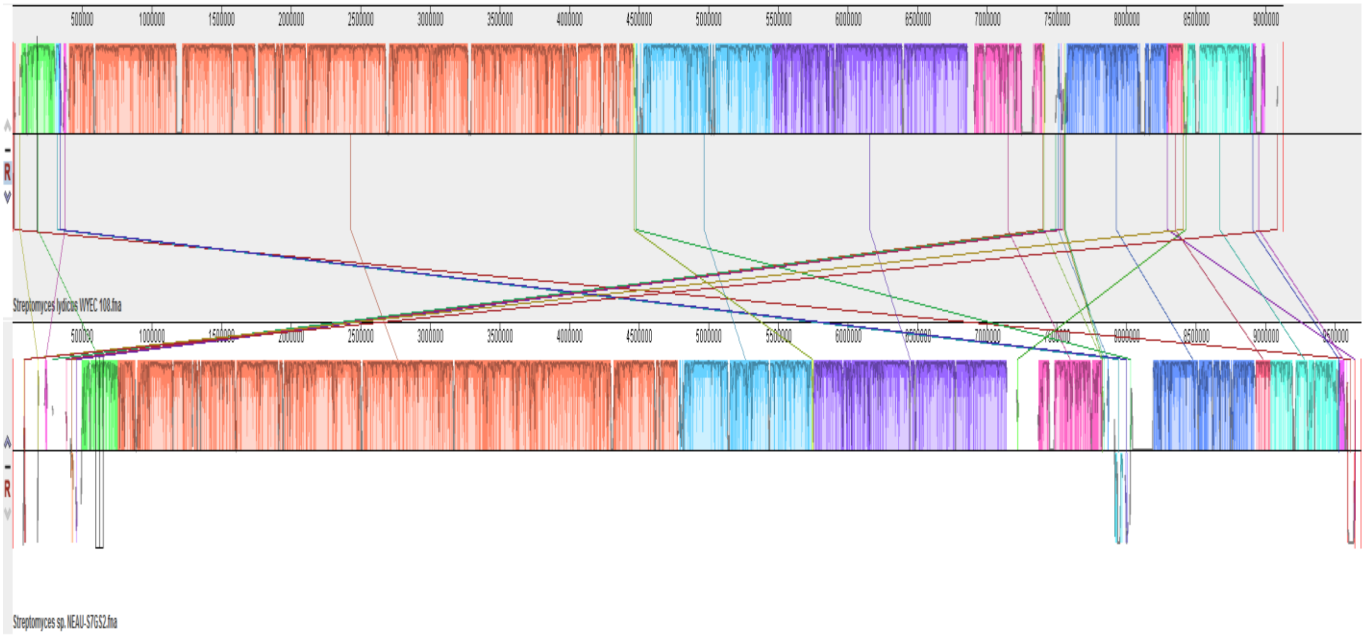

Supplementary Figure S23. Multiple genome alignment performed with the progressiveMauve algorithm for *S. lydicus* WYEC 108 and *Streptomyces* sp. NEAU-S7GS2.

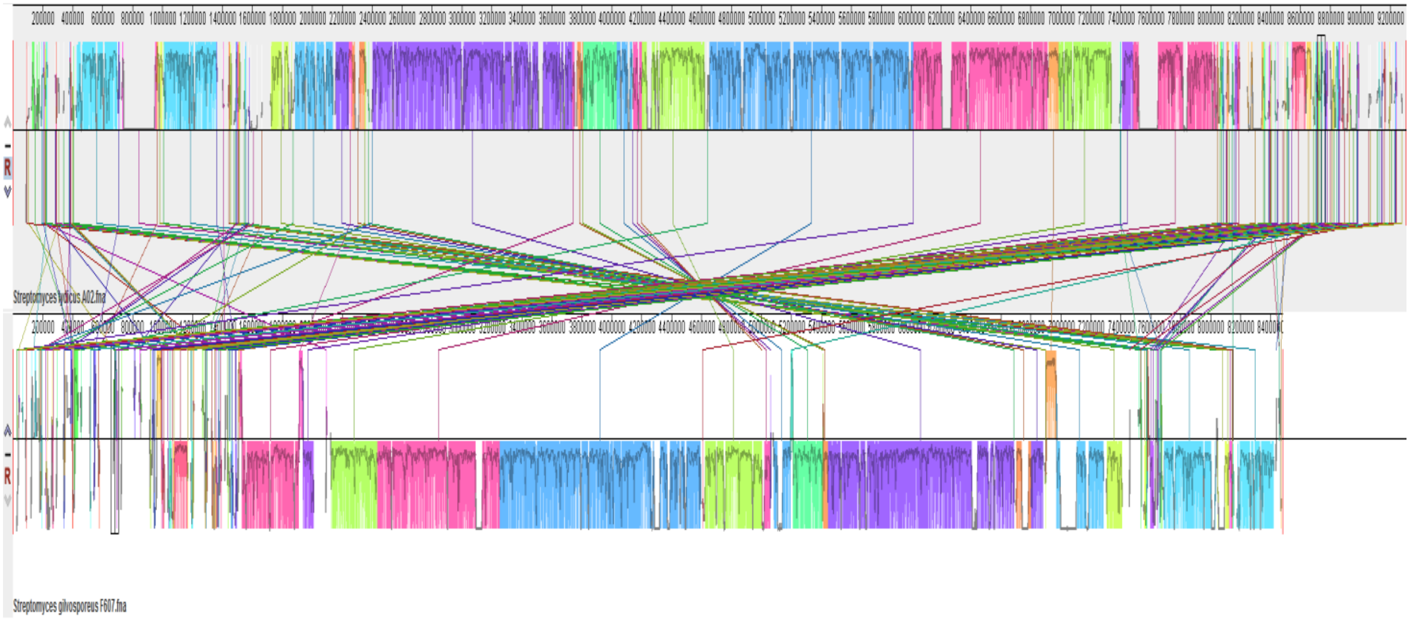

Supplementary Figure S24. Multiple genome alignment performed with the progressiveMauve algorithm for *S. lydicus* A02 and *S. gilvosporeus* F607.

.

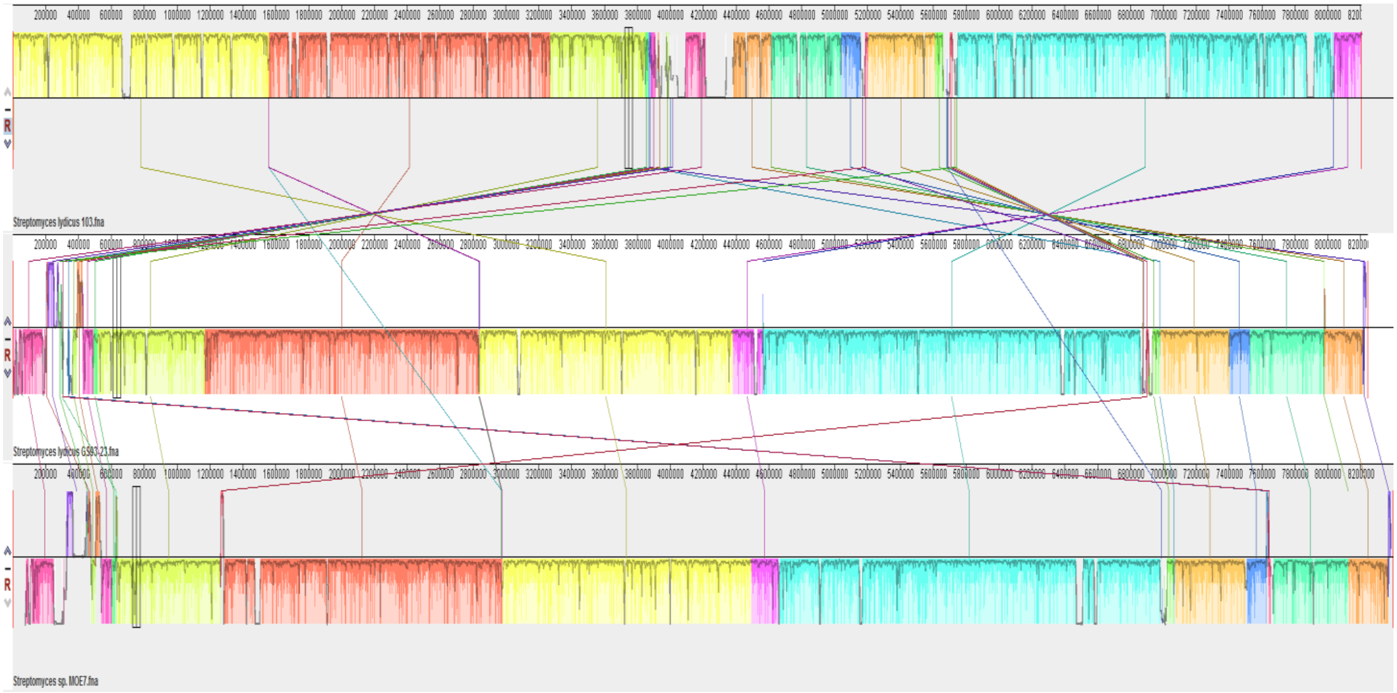

Supplementary Figure S25. Multiple genome alignment performed with the progressiveMauve algorithm for *S. lydicus* 103, *S. lydicus* GS93 23 and *Streptomyces* sp. MOE7.

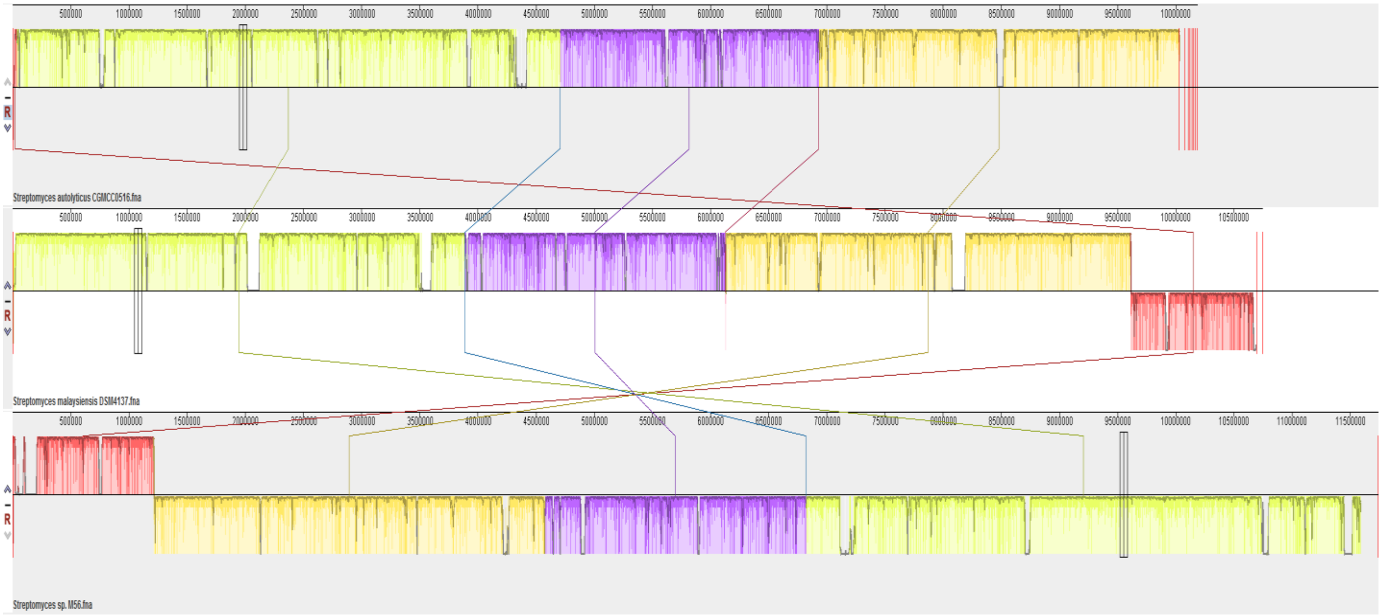

Supplementary Figure S26. Multiple genome alignment performed with the progressiveMauve algorithm for *S. autolyticus* CGMCC0516, *S. malaysiensis* DSM 4137 and *Streptomyces* sp. M56.

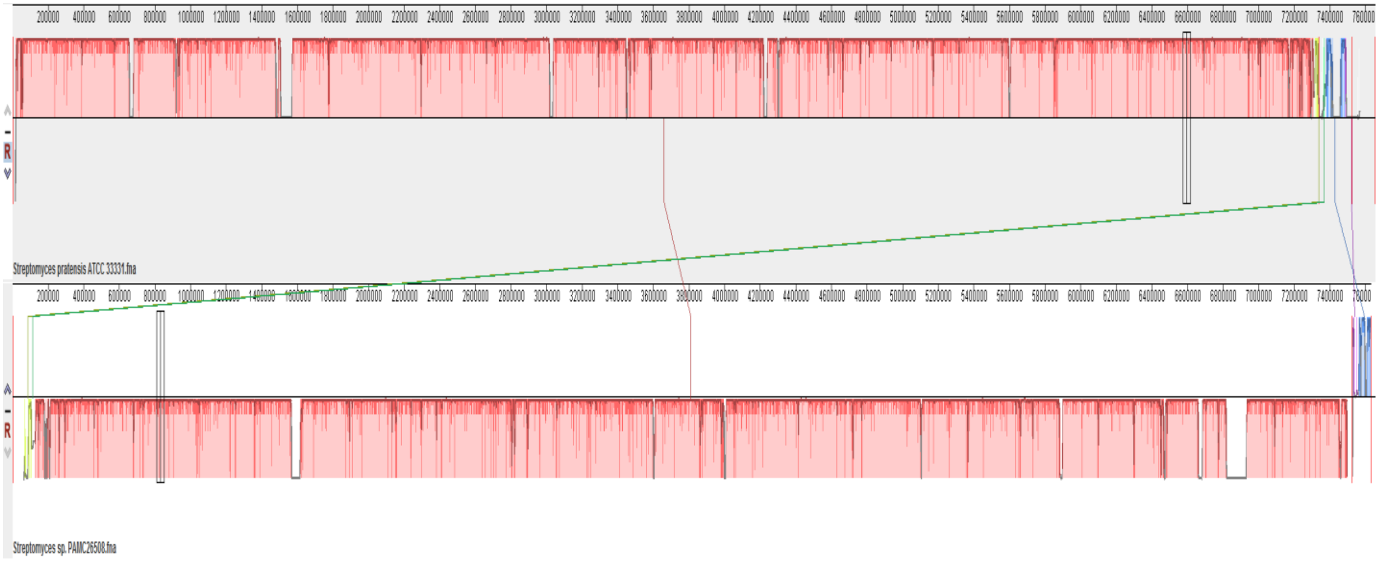

Supplementary Figure S27. Multiple genome alignment performed with the progressiveMauve algorithm for *S. pratensis* ATCC 33331 and *Streptomyces* sp. PAMC26508.

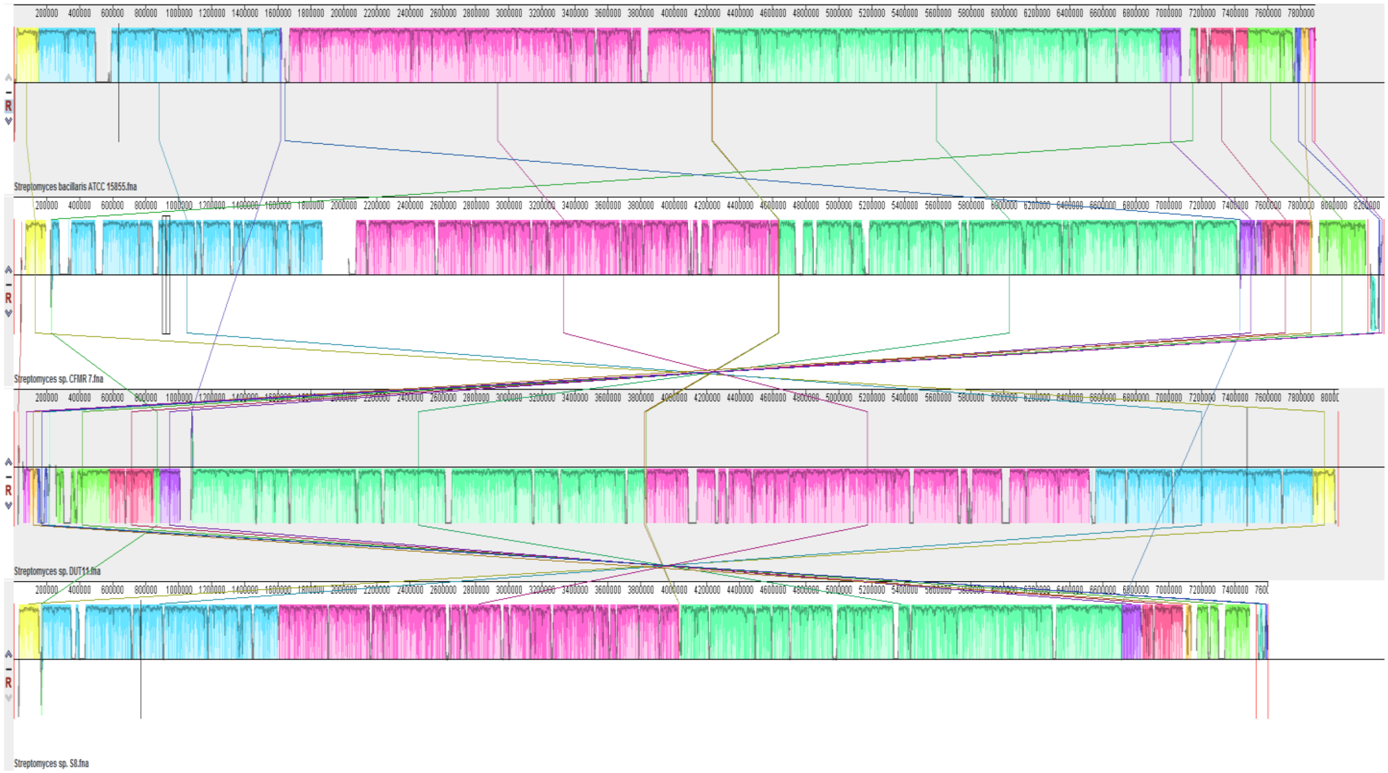

Supplementary Figure S28. Multiple genome alignment performed with the progressiveMauve algorithm for *S. bacillaris* ATCC 15855, *Streptomyces* sp. DUT11, *Streptomyces* sp. CFMR7 and *Streptomyces* sp. S8.

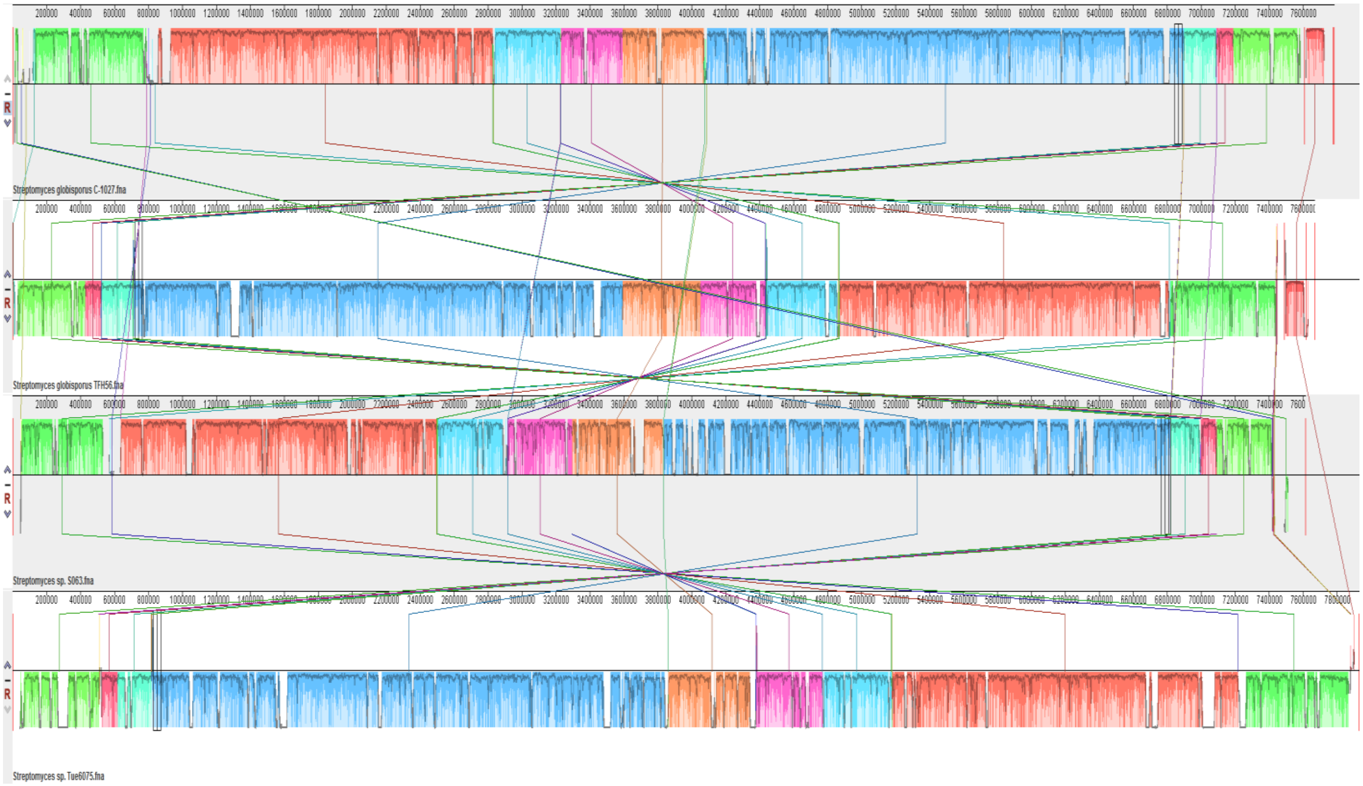

Supplementary Figure S29. Multiple genome alignment performed with the progressiveMauve algorithm for *S. globisporu*s C-1027, *S. globisporus* TFH56, *Streptomyces* sp. 6063 and *Streptomyces* sp. Tue6075.

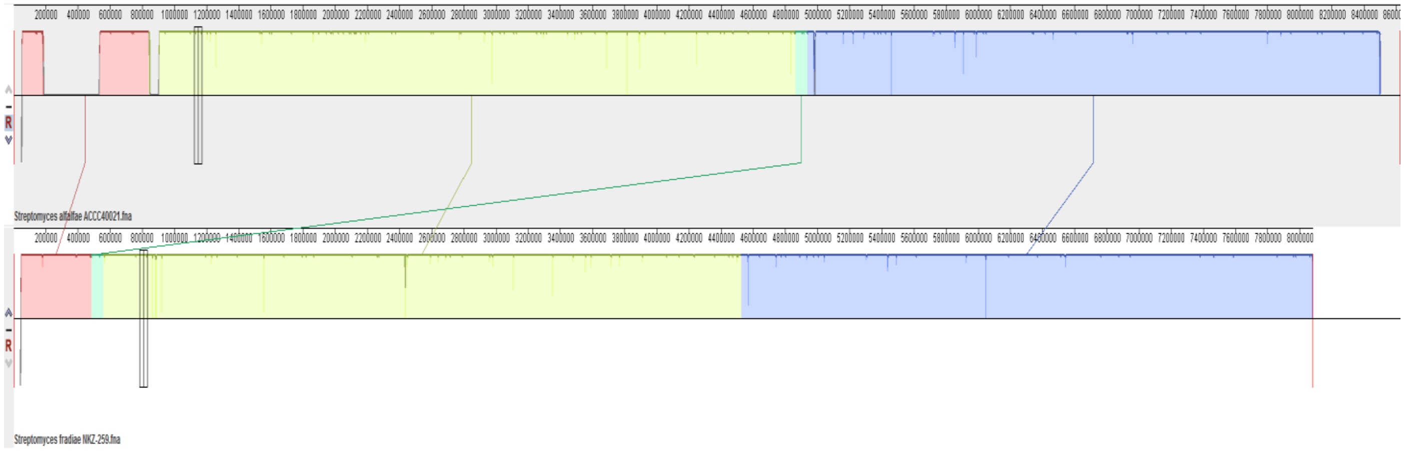

Supplementary Figure S30. Multiple genome alignment performed with the progressiveMauve algorithm for *S. fradiae* NKZ-259 and S. *alfalfae* ACCC40021.

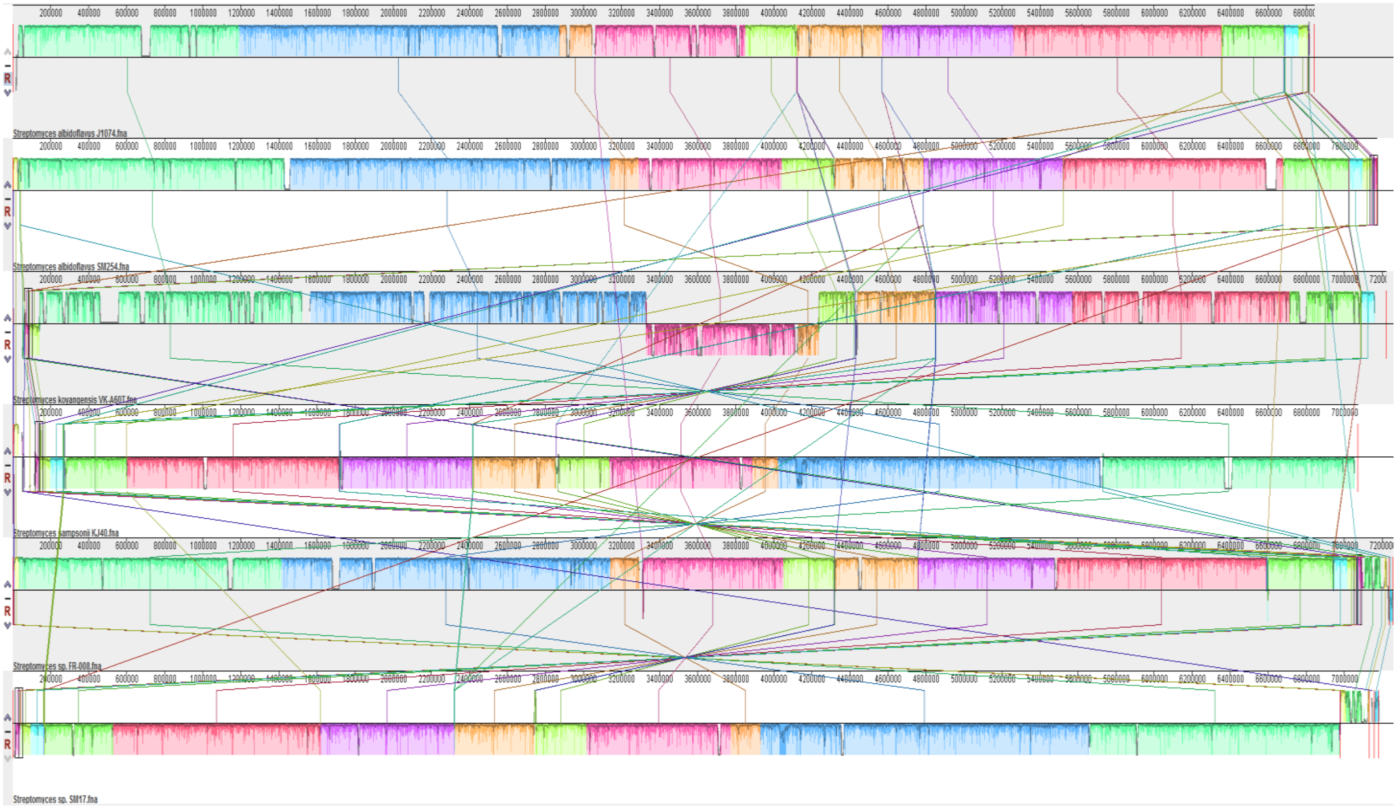

Supplementary Figure S31. Multiple genome alignment performed with the progressiveMauve algorithm for *S. koyangensis* VK-A60T, *S. sampsonii* KJ40, and *Streptomyces* sp. Fr-008, *S. albidoflavus* J1074, *S. albidoflavus* SM254 and *Streptomyces* sp. SM17.
